# Protocol for generating human assembloids to investigate thalamocortical and corticothalamic synaptic transmission and plasticity

**DOI:** 10.1016/j.xpro.2025.103630

**Published:** 2025-02-07

**Authors:** Anjana Nityanandam, Mary H. Patton, Ildar T. Bayazitov, Kyle D. Newman, Kristen T. Thomas, Stanislav S. Zakharenko

**Affiliations:** 1 Department of Developmental Neurobiology, St. Jude Children’s Research Hospital, Memphis, TN 38105, USA

**Keywords:** Neuroscience, Stem Cells, Organoids

## Abstract

Human induced pluripotent stem cells (hiPSCs) can be used to generate assembloids that recreate thalamocortical circuitry displaying short-term and long-term synaptic plasticity. Here, we describe a protocol for differentiating hiPSCs into thalamic and cortical organoids and then fusing them to generate thalamocortical assembloids. We detail the steps for using whole-cell patch-clamp electrophysiology to investigate the properties of synaptic transmission and synaptic plasticity in this model system.

For complete details on the use and execution of this protocol, please refer to Patton et al.^1^

## Highlights

• Procedure for the guided differentiation of hiPSCs into thalamic and cortical organoids
• Instructions for generating thalamocortical assembloids
• Steps for whole-cell patch-clamp electrophysiology on thalamocortical assembloids

## Abstract (editor highlights)

Publisher’s note: Undertaking any experimental protocol requires adherence to local institutional guidelines for laboratory safety and ethics.

## Graphical abstract

**Figure:**
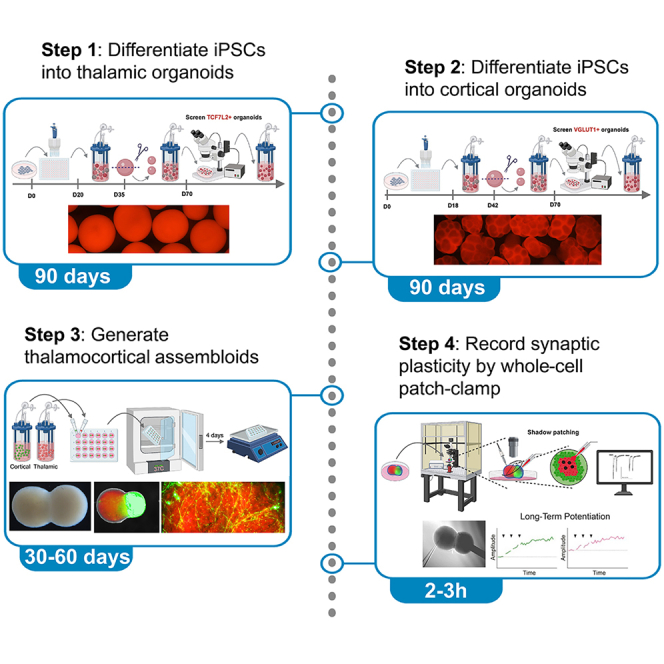

## Before you begin

Human iPSCs have the potential to differentiate into three-dimensional self-organizing structures called organoids that can partially recapitulate physiological and cytoarchitectural features of specific regions of the human brain.^2^^,^^3^^,^^4^^,^^5^ By exposing hiPSCs to a combination of small molecules at specific concentrations in a temporally defined manner, they can be coerced towards brain region–specific lineages. Once committed, they can be made to differentiate along a developmentally relevant pathway into organoids composed of mature neurons and glia specific to those brain regions. Organoids modeling two different brain regions can then be fused to generate larger, more complex structures called assembloids.^6^^,^^7^^,^^8^^,^^9^^,^^10^^,^^11^ In this model system, neurons from either side of the assembloid will extend long-range projections towards each other and establish reciprocal synaptic connections, thereby establishing the rudimentary circuitry reminiscent of that which exists between the two brain regions *in vivo*.^8^^,^^12^^,^^13^^,^^14^

Here, we provide a step-by-step procedure for differentiating hiPSCs into thalamic and cortical organoids then fusing them to create thalamocortical assembloids. We adapted and modified multiple methods to optimize our own protocol for generating mature cortical organoids.^15^^,^^16^^,^^17^ Similarly, we used our understanding of thalamic development in mice, acquired through previous developmental biology studies,^18^^,^^19^^,^^20^^,^^21^^,^^22^^,^^23^ and adapted two published PSC-derived thalamic model systems to optimize our own protocol for generating mature thalamic organoids from hiPSCs.^13^^,^^24^ Although thalamocortical assembloids have been generated by others,^11^^,^^13^^,^^25^ our study is the first to generate thalamic organoids consisting mostly of excitatory thalamic neurons and the first to demonstrate synaptic plasticity in this model system by using whole-cell patch-clamp electrophysiology.^1^

We also detail the use of tissue-specific reporter lines to identify and select high-quality organoids for assembloid creation. Specifically, we recommend using a fluorescent protein (e.g., tdTomato) under the control of a thalamic lineage–specific promoter, such as *TCF7L2*, to generate a thalamic reporter hiPSC line.^26^^,^^27^ We also recommend using a fluorescent protein (e.g., tdTomato) under the control of a cortical lineage–specific neuronal promoter, such as *VGLUT1*, to generate a cortical reporter hiPSC line.^28^^,^^29^ Using reporter lines helped us to optimize the health, long-term survival, and functional maturation of these organoids. Next, we describe the steps for using individual thalamic and cortical organoids to build an assembloid model system that can recapitulate, at least partially, the basic features of the thalamocortical circuitry. Finally, we describe the protocol for performing whole-cell patch-clamp recordings with the assistance of the two-photon “shadow” approach,^30^^,^^31^ which facilitates single-cell electrophysiological recordings. In our recently published study,^1^ we used this protocol to demonstrate that the thalamocortical circuitry within these assembloids displays short-term and long-term synaptic plasticity at both thalamocortical and corticothalamic synapses.

### Institutional permissions

The use of hiPSCs to generate organoids was approved by the St. Jude Institutional Review Board. Authors involved in the direct handling of hiPSCs were required to obtain completion certificates for the CITI programs “Human Subjects Research” and “Human Embryonic Stem Cell Research.”

All researchers should obtain the required approvals and permissions before initiating any work with hiPSCs.

### Validate parent hiPSC lines before generating lineage-specific reporters

**Timing: 1–2 months**

iPSC lines often carry genetic abnormalities that can affect the results of downstream assays.^32^^,^^33^^,^^34^^,^^35^^,^^36^ In addition, interline variability between multiple hiPSC clones derived from the same donor cell population is common.^37^^,^^38^ Therefore, we recommend analyzing at least three clones per donor and selecting the best-quality clone for reporter generation and differentiation. To ensure optimal efficiency of organoid generation, we recommend performing the following well-established assays on hiPSC lines: 1) karyotyping by G-banding to ensure genomic integrity; 2) immunostaining for pluripotency markers such as NANOG, OCT4, SOX2, SSEA4, TRA-1-60, and TRA-1-81; 3) RT-qPCR screening for pluripotency markers such as *DNMT3B, ZFP42, TDGF1, GDF3,* and *LIN28A*; 4) qPCR-based copy number variation (CNV) analysis at the eight most frequently aberrant chromosomal locations^39^^,^^40^; and 5) a trilineage differentiation assay to confirm the ability of the hiPSC line to generate all three germ lineages.^41^^,^^42^^,^^43^ These recommendations are in accordance with the “Standards for Stem Cell Use in Research” proposed by the International Society for Stem Cell Research.^44^ In addition, hiPSCs should be checked for *Mycoplasma* contamination prior to reporter generation, as well as during and after differentiation. We also recommend checking the DNA methylation status at select epigenetic markers of pluripotency, for example, by analyzing the line on an Illumina Methylation EPIC v2.0 BeadChip. A high-quality hiPSC line is expected to show hypomethylation at pluripotency-associated genes such as *DPPA4, SALL2, SALL4, EPHA1,* and *RAB25*, in addition to those listed above, and hypermethylation at somatic genes such as *GBP4, SP100, DCN, GPNMB, FAP, MMP3, MMP1,* and *GBP3*.^1^^,^^45^^,^^46^

***Note:*** Optical genome mapping can be performed as an alternative to G-banding and has the added advantage of providing much higher resolution. As an alternative to the qPCR-based CNV assay, lines can be analyzed on a whole-genome, SNP-based genotyping microarray, which provides higher resolution and more accurate evaluation of the genomic integrity of the line.

### Generate thalamic lineage-specific (TCF7L2) and cortical lineage-specific (VGLUT1) reporter lines

**Timing: 3–4 months**

The purpose of generating reporter lines is to screen the organoid population and select high-quality thalamic and cortical organoids for creating assembloids suitable for optical or electrophysiological experiments. Most organoid differentiation protocols are less than 100% efficient and often generate organoids of variable quality.^47^^,^^48^ Because of this, using reporter lines can help improve the success and efficiency of the overall experiment.

We used the CRISPR-Cas9 approach to generate thalamic lineage–specific and cortical lineage–specific reporter lines. For the thalamic reporter, we inserted the *tdTomato (tdT)* coding sequence into the N-terminal of the endogenous *TCF7L2* coding sequence separated by the sequence encoding the self-cleaving peptide P2A. We chose to tag the N-terminal because TCF7L2 has multiple isoforms that differ in their C-terminal ends, but have the same N- terminal. To generate the cortical reporter, we inserted the *tdT* coding sequence into the C-terminus of the endogenous *SLC17A7 (VGLUT1)* coding sequence separated by coding sequence encoding the self-cleaving peptide P2A. We chose to tag the C-terminus because we reasoned that it is less likely to affect targeting to the ER during synthesis. In addition, we found examples in literature of mouse lines where VGLUT1 was tagged at the C-terminus.^49^ Because hiPSC lines have variable inherent biases in their differentiation potential, it is best to generate all reporter lines from multiple donors, who will have different genetic backgrounds.

***Note:*** The hiPSC line used in this study, TP-190a, was reprogrammed from dental pulp cells obtained from a healthy male subject. The reprogramming was performed with episomal plasmids at ALSTEM, CA. The dental pulp cells were provided by Dr. Lawrence Reiter of Tulane University.

***Note:*** Before proceeding with reporter generation, we analyzed three clones of the TP-190a parent hiPSC line and selected the clone that was karyotypically normal, the colonies of which were densely (tightly) packed with no signs of spontaneous differentiation and generated 2D neural rosettes with high efficiency. We performed the neural rosette formation assay to assess the neurogenic potential of each clone. Similarly, we analyzed multiple clones of the TP-190a-TCF7L2-tdT and TP-190a-VGLUT1-tdT reporter hiPSC lines and selected the clones that had a normal karyotype and that generated densely (tightly) packed colonies with no spontaneous differentiation. The selected clones were thoroughly validated using the aforementioned assays, the results of which are reported in the published article.

***Alternatives:*** Reporter expression will be needed to screen the organoid population, but any fluorescent reporter (not necessarily tdTomato) can be used to generate the TCF7L2 and VGLUT1 reporter lines as long as the fluorescence stereomicroscope has the appropriate filter.

### Prepare aliquots of hSyn-GFP lentiviral vector

**Timing: 2 weeks**

To visualize neurons in VGLUT1-tdTomato^+^ cortical organoids and to identify the cortical organoid within thalamocortical assembloids, we recommend using lentiviral vectors that express a fluorescence reporter (e.g., eGFP) under the control of the human synapsin promoter (hSyn). To this end, the St. Jude Vector Core generated lentiviral vectors with the VSV-G pseudotype, using the pHR-hSyn-eGFP (hSyn-GFP) lentiviral plasmid from Addgene.

### Thaw, plate, and grow hiPSCs in culture to 80% confluency

**Timing: 1 h (for step 1)**

**Timing: 30 min (for step 2)**

**Timing: 3–4 days (for step 3)**

1. Coat a 6-well plate with hES-qualified Matrigel.
  a. Thaw one 250-μL aliquot of hES-Matrigel for 16–18 h at 4°C.
  b. The next day, prepare Matrigel coating solution by diluting 250 μL of thawed Matrigel in 25 mL of DMEM:F12 on ice.
  c. Mix thoroughly by gently flipping the tube multiple times.
  d. Coat one 6-well plate with 1 mL of Matrigel coating solution per well.
  e. Leave the plate at 22°C for 1 h to complete the coating.
  f. Wash the plate with 2 mL per well of DMEM:F12 warmed to 22°C .

***Note:*** If needed, Matrigel-coated plates can be left in the incubator for up to 3 days before cells are plated.

2. Thaw and plate a cryovial of hiPSCs.
  a. Warm sterile water in a sterile beaker placed inside a bead bath set to 37°C.
  b. Warm 20 mL of mTeSR1 supplemented with 2 μM Thiazovivin in a bead bath set to 37°C.
  c. Remove a cryovial from liquid nitrogen and place it on dry ice for transport to the laboratory.
  d. Place the cryovial in a floater and place the floater on the warmed water in the bead bath for 1 min 40 s.
  e. Remove the cryovial from the bead bath while a blob of ice remains inside.
  f. Spray the cryovial with 70% ethanol to sterilize it before bringing the vial inside the biosafety cabinet (BSC).
  g. Uncap the vial and using a 5-mL serological pipette, add 1 mL of the warm mTeSR1+thiazovivin medium.
  h. Aspirate the cells from the cryovial back into the same serological pipette and transfer the entire suspension directly into 8 mL of mTeSR1.
  i. Centrifuge the tube at 300 × *g* for 4 min.
  j. Aspirate the supernatant. Tap the pellet to resuspend it as a homogeneous suspension.
  k. Add 12 mL of mTeSR1+thiazovivin to the cell suspension, mix well, and plate 2 mL of this cell suspension in each well of the Matrigel-coated 6-well plate.
  l. Swirl the plate twice (using a circular motion), then move it up and down twice then side to side twice. This will ensure a homogeneous distribution of cells over the entire surface area of the well.
  m. Place the plate in a hypoxic incubator at 37°C, 5% CO_2_, and 5% O_2_.

***Note:*** hiPSCs can also be grown in culture in normoxic (20% O_2_) conditions, but there is evidence that growing them in low-oxygen conditions reduces the rate of the mutations that they tend to acquire during growth in culture and passaging.^50^

3. Grow hiPSCs in culture to 70%–80% confluency.
  a. The day after plating, remove the plate from the incubator and swirl it to collect the floating dead cells.
  b. Aspirate the spent medium from each well by using a vacuum line and a sterile, unfiltered P1000 tip.
  c. Add 2 mL of mTeSR1 warmed to 22°C to each well.
  d. Observe the cells under the Stemi508 to determine their confluency and under the AxioObserver to check their morphology and confirm the absence of spontaneous differentiation.
  e. Repeat the above steps every day until the cells reach 70%–80% confluency.

## Key resources table

**Table:** 

REAGENT or RESOURCE	SOURCE	IDENTIFIER
**Bacterial and virus strains**		

pHR-hSyn-eGFP	Addgene	114215

**Chemicals, peptides, and recombinant proteins**		

Insulin	Sigma-Aldrich	I9278-5ML
SB431542	Tocris	1614
Dorsomorphin	Tocris	3093
Thiazovivin	Tocris	3845
Growth factor reduced Matrigel	Corning	354230
FGF8b	PeproTech	100–25
SAG (Smoothened agonist)	STEMCELL Technologies	73412
IWR1e (WNT inhibitor)	Sigma-Aldrich	681669-10MG
MEK inhibitor	R&D Systems	S1036
BMP7	R&D Systems	354-BP-010
N2 supplement	Life Technologies	17502048
B27 supplement without vitamin A	Life Technologies	12587010
Heat-stable bFGF	Gibco	PHG0369
EGF	R&D Systems	236EG200
BDNF	PeproTech	450-02-50UG
GDNF	PeproTech	450-10-50UG
ES-qualified FBS	Life Technologies	10439024
DAPT	R&D Systems	2634/10
Ascorbic acid	Sigma-Aldrich	A4403-100MG
Dibutyryl cAMP	Sigma-Aldrich	D0627-100MG
Cyclopamine	STEMCELL Technologies	72074
BrainPhys	STEMCELL Technologies	05790
Knockout Serum Replacement	Thermo Fisher Scientific	10828028
GlutaMAX	Thermo Fisher Scientific	35050061
Non-essential amino acids	Thermo Fisher Scientific	11140050
2-Mercaptoethanol	Sigma-Aldrich	M3148-100ML
Antibiotic-Antimycotic	Thermo Fisher Scientific	15240062
Glasgow’s minimum essential medium (GMEM)	Sigma-Aldrich	G5154
DMEM:F12	Thermo Fisher Scientific	11330032
Chemically defined lipid concentrate	Thermo Fisher Scientific	11905031
Heparin	Sigma-Aldrich	H3149-10KU
Ham’s F12	Lonza	12–615F
IMDM	Thermo Fisher Scientific	1244053
Monothioglycerol	Sigma-Aldrich	M6145
Apotransferrin	Sigma-Aldrich	T1428
20% BSA	Fisher Scientific	50-255-465
NaCl	Fisher Chemical	S271-3
KCl	Sigma-Aldrich	P9333
CaCl_2_	Carolina	85-1845
MgCl_2_	Honeywell	63020
NaH_2_PO_4_	Sigma-Aldrich	S8282
NaHCO_3_	Sigma-Aldrich	S8875
D-glucose	Sigma-Aldrich	G7528
CsMeSO_3_	Sigma-Aldrich	C1426
CsCl	Sigma-Aldrich	C4036
HEPES	Sigma-Aldrich	H3784
EGTA	Sigma-Aldrich	E3889
ATP-Mg_2_	Sigma-Aldrich	A9187
GTP-Na	Sigma-Aldrich	G8877
Phosphocreatine-Na_2_	Sigma-Aldrich	P7936
QX-314 chloride	Hello Bio	HB1030
TEA-Cl	Sigma-Aldrich	T2265
1 N hydrochloric acid	Sigma-Aldrich	H9892
Alexa Fluor 594	Invitrogen	A10442

**Critical commercial assays**		

STEMdiff Trilineage Differentiation Kit	STEMCELL Technologies	05230
StemLight Pluripotency Antibody Kit	Cell Signaling	9656S
hPSC Genetic Analysis Kit	STEMCELL Technologies	07550

**Experimental models: Cell lines**		

Human iPSC TP-190a-TCF7L2-tdTomato	CAGE, St Jude	
Human iPSC TP-190a-VGLUT1-tdTomato	CAGE, St Jude	

**Software and algorithms**		

Electrophysiology DAQ and analysis software	Axon Instruments	pClamp 10.7

**Other**		

Low-attachment 96V plates	SBio	MS-9096VZ
Magnetic stir (bioreactor) unit	ABLE Biott	ABBWBP03N0S
Disposable bioreactor units	ABLE Biott	ABBWVS03A
Low-attachment 24-well plate	Corning	3473
Automated cell counter	Logos Biosystems	LUNA-II (L40002)
Fluorescence microscope	Zeiss	Axio Observer.D1
Fluorescence stereomicroscope	Zeiss	SteREO Discovery.V12
Stereomicroscope	Zeiss	Stemi 508
Camera and controller unit for stereomicroscope	Nikon	DS-Fi2/DS-L3
Orbital shaker	Benchmark Scientific	BT302
Ultra Fine clipper scissors	Fine Science Tools	15300-00
Osmometer	Fiske	Model 210
Microscope	Olympus	BX51WI
Motorized stage	Bruker	Custom
Two-photon imaging system	Bruker	Custom
Laser	Coherent	Chameleon
Amplifier	Axon Instruments	Multiclamp 700B
Digitizer	Axon Instruments	Digidata 1550B
Stimulus isolator	A.M.P.I.	ISO-Flex
Bipolar concentric stimulation electrode	FHC Inc.	CBBPE75 (custom)
Stainless steel wire	McMaster-Carr	6517K68
Male pins	World Precision Instruments	5482
Solder/soldering iron	Various sources	
Heat-shrink tubing	Various sources	
Heat gun	Various sources	
Chamber heater	Warner Instruments	CL-100
Micromanipulator	Luigs & Neumann	SM-5
Pipette puller	Sutter	P-2000
Carbogen	NexAir	UN3156
Electrophysiology slice anchor	Warner Instruments	WI 64-1420
Plastic transfer pipettes	Fisherbrand	13-711-7M
Borosilicate glass pipettes	Sutter	BF150-86-10
Incubator	Thermo Scientific	HERAcell 150i
Centrifuge	Thermo Scientific	Sorvall ST 8R
Bead sterilizer	CellPoint	Germinator 500 (Model#39GF)
Laminar flow hood	Thermo Scientific	HeraGuard ECO
Biological safety cabinet	Baker	SterilGuard e3 (Model#SG404)

## Materials and equipment

**Equipment**: This protocol requires common tissue culture and electrophysiology supplies and equipment, as listed above.

***Note:*** All tissue culture media should be filtered using a membrane with 0.22-μm pores. We suggest preparing a 250 mL–500 mL stock of each medium, but do not freeze them. Once thawed, small molecules can be stored at 4°C for up to 1 month. Small molecules can be added to the stock media and stored at 4°C for up to 1 month, unless otherwise specified.

***Alternatives:*** Any stereomicroscope with a dissecting stage can be used in place of the Zeiss Stemi 508 to observe EBs and organoids.

Any fluorescence stereomicroscope equipped with appropriate filters to detect tdTomato and eGFP can be used in place of the Zeiss Discovery.V12 to screen organoid populations.

Cell counting can be done manually using a hemocytometer instead of on an automated cell counter.

As an alternative to the ABLE Biott magnetic stirrer units, any bioreactor-like system that can maintain organoids in a gentle but constant state of agitation while minimizing fusion of single organoids into larger structures can be used, but the protocol might require additional optimization.

**Table:** Cortical EB Medium (CEM)

Reagent	Final concentration	Amount	Storage
DMEM:F12	N/A	185 mL	4°C
Knockout Serum Replacement ∗Do not filter∗	20%	50 mL	−20°C
ES-FBS ∗Do not filter∗	3%	7.5 mL	−20°C
GlutaMAX (100×)	1×	2.5 mL	22°C
Non-Essential Amino Acids (100×)	1×	2.5 mL	4°C
2-Mercaptoethanol (1000×)	1×	250 μL	4°C
Antibiotic-Antimycotic (100×)	1×	2.5 mL	−20°C
**Total**	**N/A**	**250 mL**	**4°C**
SB431542 (10 mM)	5 μM	10 μL for 20 mL	−80°C
Dorsomorphin	2 M	20 μL for 20 mL	−80°C
IWR1e (WNT inhibitor) (6 mM)	3 μM	10 μL for 20 mL	−20°C
Thiazovivin (4 mM)	2 μM	10 μL for 20 mL	−20°C
GFR-Matrigel (add the day before plating and leave at 4°C)	1% v/v	200 μL for 20 mL	−20°C

Store at 4°C for up to 1 month.

**Table:** Cortical Specification Medium (CSM)

Reagent	Final concentration	Amount	Storage
Glasgow’s Minimum Essential Medium (GMEM)	N/A	192 mL	4°C
Knockout Serum Replacement ∗Do not filter∗	20%	50 mL	−20°C
Non-Essential Amino Acids (100×)	1×	2.5 mL	4°C
2-Mercaptoethanol (500×)	1×	500 μL	4°C
Antibiotic-Antimycotic (100×)	1×	2.5 mL	−20°C
**Total**	**N/A**	**250 mL**	**4°C**
SB431542 (10 mM)	5 μM	10 μL for 20 mL	−80°C
IWR1e (WNT inhibitor) (6 mM)	3 μM	10 μL for 20 mL	−20°C
Cyclopamine (5 mM)	2.5 μM	10 μL for 20 mL	−20°C
Thiazovivin (4 mM)	2 μM	10 μL for 20 mL	−20°C

Store at 4°C for up to 1 month.

**Table:** Cortical Neural Induction Medium (CNIM)

Reagent	Final concentration	Amount	Storage
Glasgow’s Minimum Essential Medium (GMEM)	N/A	192 mL	4°C
Knockout Serum Replacement ∗Do not filter∗	20%	50 mL	−20°C
Non-Essential Amino Acids (100×)	1×	2.5 mL	4°C
2-Mercaptoethanol (500×)	1×	500 μL	4°C
Antibiotic-Antimycotic (100×)	1×	2.5 mL	−20°C
**Total**	**N/A**	**250 mL**	**4°C**
SB431542 (10 mM)	5 μM	10 μL for 20 mL	−80°C
IWR1e (WNT inhibitor) (6 mM)	3 μM	10 μL for 20 mL	−20°C

Store at 4°C for up to 1 month.

**Table:** Cortical Neural Differentiation Medium I (CNDM I)

Reagent	Final concentration	Amount	Storage
DMEM:F12	N/A	242.5 mL	4°C
Chemically Defined Lipid Concentrate ∗Do not filter∗ (100×)	1×	2.5 mL	4°C
N2 Supplement ∗Do not filter∗ (100×)	1×	2.5 mL	−20°C
Antibiotic-Antimycotic (100×)	1×	2.5 mL	−20°C
B27 without vitamin A (50×) ∗Do not filter∗	1×	5 mL	−20°C
**Total**	**N/A**	**250 mL**	**4°C**
bFGF (20 mg/ mL)	20 ng/mL	20 μL for 20 mL	−20°C
EGF (10 μg/mL)	20 ng/mL	40 μL for 20 mL	−20°C

Store at 4°C for up to 1 month.

**Table:** Cortical Neural Differentiation Medium II (CNDM II)

Reagent	Final concentration	Amount	Storage
DMEM:F12	N/A	237.5 mL	4°C
Chemically Defined Lipid Concentrate ∗Do not filter∗ (100×)	1×	2.5 mL	4°C
N2 Supplement ∗Do not filter∗ (100×)	1×	2.5 mL	−20°C
Antibiotic-Antimycotic (100×)	1×	2.5 mL	−20°C
B27 without vitamin A (50×) ∗Do not filter∗	1×	5 mL	−20°C
**Total**	**N/A**	**250 mL**	**4°C**

Store at 4°C for up to 1 month.

**Table:** Cortical Neural Differentiation Medium III (CNDM III)

Reagent	Final concentration	Amount	Storage
DMEM:F12	N/A	212.5 mL	4°C
N2 Supplement ∗Do not filter∗ (100×)	1×	2.5 mL	−20°C
B27 without vitamin A (50×) ∗Do not filter∗	1×	5 mL	−20°C
Chemically Defined Lipid Concentrate ∗Do not filter∗ (100×)	1×	2.5 mL	4°C
Antibiotic-Antimycotic (100×)	1×	2.5 mL	−20°C
ES FBS ∗Do not filter∗	10%	25 mL	−20°C
Heparin	5 μg/mL	125 μL	−20°C
**Total**	**N/A**	**250 mL**	**4°C**

Store at 4°C for up to 1 month.

**Table:** Cortical Neural Maturation Medium I (CNMM I)

Reagent	Final concentration	Amount	Storage
DMEM:F12	N/A	217.5 mL	4°C
N2 Supplement ∗Do not filter∗ (100×)	1×	2.5 mL	−20°C
Chemically Defined Lipid Concentrate ∗Do not filter∗ (100×)	1×	2.5 mL	4°C
Antibiotic-Antimycotic (100×)	1×	2.5 mL	−20°C
ES FBS ∗Do not filter∗	10%	25 mL	−20°C
Heparin	5 μg/mL	125 μL	−20°C
**Total**	**N/A**	**250 mL**	**4°C**
B27 without vitamin A (50×) ∗Do not filter∗	1×	5 mL for 250 mL	−20°C
BDNF (10 μg/mL)	10 ng/mL	20 μL for 20 mL	−80°C
GDNF (10 μg/mL)	10 ng/mL	20 μL for 20 mL	−80°C

Store at 4°C for up to 1 month.

**Table:** Cortical Neural Maturation Medium II (CNMM II)

Reagent	Final concentration	Amount	Storage
BrainPhys	N/A	215 mL	4°C
ES FBS ∗Do not filter∗	10%	25 mL	−20°C
Antibiotic-Antimycotic (100×)	1×	2.5 mL	−20°C
N2 Supplement ∗Do not filter∗ (100×)	1×	2.5 mL	−20°C
B27 without vitamin A ∗Do not filter∗ (50×)	1×	5 mL	−20°C
**Total**	**N/A**	**250 mL**	**4°C**
BDNF (10 μg/mL)	10 ng/mL	20 μL for 20 mL	−80°C
GDNF (10 μg/mL)	10 ng/mL	20 μL for 20 mL	−80°C
Thiazovivin (4 mM)	2 μM	10 μL for 20 mL	−20°C
GFR-Matrigel	1% v/v	200 μL for 20 mL	−20°C

Store at 4°C for up to 1 month.

**Table:** Cortical Neural Maturation Medium III (CNMM III)

Reagent	Final concentration	Amount	Storage
BrainPhys	N/A	234 mL	4°C
ES FBS ∗Do not sterile filter∗	1%	2.5 mL	−20°C
N2 Supplement ∗Do not filter∗ (100×)	1×	2.5 mL	−20°C
B27 without vitamin A ∗Do not filter∗ (50×)	1×	5 mL	−20°C
GlutaMAX (100×)	1×	2.5 mL	22°C
Non-Essential Amino Acids (100×)	1×	2.5 mL	4°C
Ascorbic Acid	200 μM	1 mL	4°C
**Total**	**N/A**	**250 mL**	**4°C**
BDNF (10 μg/mL)	20 ng/mL	40 μL for 20 mL	−80°C
GDNF (10 μg/mL)	20 ng/mL	40 μL for 20 mL	−80°C
Thiazovivin (4 mM)	2 μM	10 μL for 20 mL	−20°C
GFR-Matrigel	1% v/v	200 μL for 20 mL	−20°C
dbCAMP (100 mM)	100 μM	20 μL for 20 mL	−20°C
DAPT (10 mM)	10 μM	20 μL for 20 mL	−20°C

Store at 4°C for up to 1 month.

**Table:** Thalamic EB Medium (TEM)

Reagent	Final concentration	Amount	Storage
Ham’s F12	N/A	116.8 mL	4°C
IMDM	N/A	116.8 mL	4°C
Chemically Defined Lipid Concentrate ∗Do not filter∗ (100×)	1×	2.5 mL	4°C
Antibiotic-Antimycotic (100×)	1×	2.5 mL	−20°C
GlutaMAX (100×)	1×	2.5 mL	22°C
Monothioglycerol	450 μM	9.78 μL	4°C
Apotransferrin	15 μg/mL	2.5 mL	−20°C
20% BSA	5 mg/mL	6.25 mL	−20°C
**Total**	**N/A**	**250 mL**	**4°C**
SB431542 (10 mM)	5 μM	10 μL for 20 mL	−80°C
Dorsomorphin (2 mM)	4 μM	40 μL for 20 mL	−80°C
Insulin (10 mg/mL)	1 μg/mL	2 μL for 20 mL	4°C
Thiazovivin (4 mM)	2 μM	10 μL for 20 mL	−20°C
GFR-Matrigel (add the day before plating and leave at 4°C)	1% v/v	200 μL for 20 mL	−20°C

Store at 4°C for up to 1 month.

**Table:** Thalamic Specification Medium (TSM)

Reagent	Final concentration	Amount	Storage
Ham’s F12	N/A	116.8 mL	4°C
IMDM	N/A	116.8 mL	4°C
Chemically Defined Lipid Concentrate ∗Do not filter∗ (100×)	1×	2.5 mL	4°C
Antibiotic-Antimycotic (100×)	1×	2.5 mL	−20°C
GlutaMAX (100×)	1×	2.5 mL	22°C
Monothioglycerol	450 μM	9.78 μL	4°C
Apotransferrin	15 μg/mL	2.5 mL	−20°C
20% BSA	5 mg/mL	6.25 mL	−20°C
**Total**	**N/A**	**250 mL**	**4°C**
SB431542 (10 mM)	5 μM	10 μL for 20 mL	−80°C
Dorsomorphin (2 mM)	2 μM	20 μL for 20 mL	−80°C
SAG (100 μM)	100 nM	20 μL for 20 mL	−20°C
Fgf8b (50 μg/mL)	20 ng/mL	8 μL for 20 mL	−80°C

Store at 4°C for up to 1 month.

**Table:** Thalamic Neural Induction Medium (TNIM)

Reagent	Final concentration	Amount	Storage
Ham’s F12	N/A	116.8 mL	4°C
IMDM	N/A	116.8 mL	4°C
Chemically Defined Lipid Concentrate ∗Do not filter∗ (100×)	1×	2.5 mL	4°C
Antibiotic-Antimycotic (100×)	1×	2.5 mL	−20°C
GlutaMAX (100×)	1×	2.5 mL	22°C
Monothioglycerol	450 μM	9.78 μL	4°C
Apotransferrin	15 μg/mL	2.5 mL	−20°C
20% BSA	5 mg/mL	6.25 mL	−20°C
**Total**	**N/A**	**250 mL**	**4°C**
SB431542 (10 mM)	5 μM	10 μL for 20 mL	−80°C
BMP7 (30 μg/mL)	30 ng/mL	20 μL for 20 mL	−80°C
MEKi (1 mM)	1 μM	20 μL for 20 mL	−80°C
SAG (100 μM)	100 nM	20 μL for 20 mL	−20°C
Fgf8b (50 μg/mL)	20 ng/mL	8 μL for 20 mL	−80°C

Store at 4°C for up to 1 month.

**Table:** Thalamic Neural Differentiation Medium I (TNDM I)

Reagent	Final concentration	Amount	Storage
DMEM:F12	N/A	217.5 mL	4°C
ES FBS ∗Do not filter∗	10%	25 mL	−20°C
N2 Supplement ∗Do not filter∗ (100×)	1×	2.5 mL	−20°C
GlutaMAX (100×)	1×	2.5 mL	22°C
Antibiotic-Antimycotic (100×)	1×	2.5 mL	−20°C
**Total**	**N/A**	**250 mL**	**4°C**
BMP7 (30 μg/mL)	30 ng/mL	20 μL for 20 mL	−80°C
MEKi (1 mM)	1 μM	20 μL for 20 mL	−80°C
SAG (100 μM)	100 nM	20 μL for 20 mL	−20°C
Fgf8b (50 μg/mL)	20 ng/mL	8 μL for 20 mL	−80°C

Store at 4°C for up to 1 month.

**Table:** Thalamic Neural Differentiation Medium II (TNDM II)

Reagent	Final concentration	Amount	Storage
DMEM:F12	N/A	217.5 mL	4°C
ES FBS ∗Do not filter∗	10%	25 mL	−20°C
N2 Supplement ∗Do not filter∗ (100×)	1×	2.5 mL	−20°C
GlutaMAX (100×)	1×	2.5 mL	22°C
Antibiotic-Antimycotic (100×)	1×	2.5 mL	−20°C
**Total**	**N/A**	**250 mL**	**4°C**
bFGF (20 μg/mL)	20 ng/mL	20 μL for 20 mL	−20°C

Store at 4°C for up to 1 month.

**Table:** Thalamic Neural Differentiation Medium III (TNDM III)

Reagent	Final concentration	Amount	Storage
DMEM:F12	N/A	212.5 mL	4°C
ES FBS ∗Do not filter∗	10%	25 mL	−20°C
N2 Supplement ∗Do not filter∗ (100×)	1×	2.5 mL	−20°C
GlutaMAX (100×)	1×	2.5 mL	22°C
Antibiotic-Antimycotic (100×)	1×	2.5 mL	−20°C
B27 without vitamin A ∗Do not filter∗ (50×)	1×	5 mL	−20°C
**Total**	**N/A**	**250 mL**	**4°C**
bFGF (20 μg/mL)	20 ng/mL	20 μL for 20 mL	−20°C
EGF (10 μg/mL)	20 ng/mL	40 μL for 20 mL	−20°C

Store at 4°C for up to 1 month.

**Table:** Thalamic Neural Maturation Medium I (TNMM I)

Reagent	Final concentration	Amount	Storage
BrainPhys	N/A	215 mL	4°C
ES FBS ∗Do not filter∗	10%	25 mL	−20°C
Antibiotic-Antimycotic (100×)	1×	2.5 mL	−20°C
N2 Supplement ∗Do not filter∗ (100×)	1×	2.5 mL	−20°C
B27 without vitamin A ∗Do not filter∗ (50×)	1×	5 mL	−20°C
**Total**	**N/A**	**250 mL**	**4°C**
BDNF (10 μg/mL)	10 ng/mL	20 μL for 20 mL	−80°C
GDNF (10 μg/mL)	10 ng/mL	20 μL for 20 mL	−80°C

Store at 4°C for up to 1 month.

**Table:** Thalamic Neural Maturation Medium II (TNMM II)

Reagent	Final concentration	Amount	Storage
BrainPhys	N/A	234 mL	4°C
ES FBS ∗Do not filter∗	1%	2.5 mL	−20°C
N2 Supplement ∗Do not filter∗ (100×)	1×	2.5 mL	−20°C
B27 without vitamin A ∗Do not filter∗ (50×)	1×	5 mL	−20°C
GlutaMAX (100×)	1×	2.5 mL	22°C
Non-Essential Amino Acids (100×)	1×	2.5 mL	4°C
Ascorbic acid	200 μM	1 mL	4°C
**Total**	**N/A**	**250 mL**	**4°C**
BDNF (10 μg/mL)	20 ng/mL	40 μL for 20 mL	−80°C
GDNF (10 μg/mL)	20 ng/mL	40 μL for 20 mL	−80°C
Thiazovivin (4 mM)	2 μM	10 μL for 20 mL	−20°C
GFR-Matrigel	1% v/v	200 μL for 20 mL	−20°C
dbCAMP (100 mM)	100 μm	20 μL for 20 mL	−20°C
DAPT (10 mM)	10 μM	20 μL for 20 mL	−20°C

Store at 4°C for up to 1 month.

**Table:** Organoid Fusion Medium (OFM)

Reagent	Final concentration	Amount	Storage
BrainPhys	N/A	224 mL	4°C
ES FBS ∗Do not filter∗	5%	12.5 mL	−20°C
N2 Supplement ∗Do not filter∗ (100×)	1×	2.5 mL	−20°C
B27 without vitamin A ∗Do not filter∗ (50×)	1×	5 mL	−20°C
GlutaMAX (100×)	1×	2.5 mL	22°C
Non-Essential Amino Acids (100×)	1×	2.5 mL	4°C
Ascorbic acid	200 μM	1 mL	4°C
**Total**	**N/A**	**250 mL**	**4°C**
BDNF (10 μg/mL)	20 ng/mL	40 μL for 20 mL	−80°C
GDNF (10 μg/mL)	20 ng/mL	40 μL for 20 mL	−80°C
Thiazovivin (4 mM)	2 μM	10 μL for 20 mL	−20°C
GFR-Matrigel (Add the day before plating and leave at 4°C)	0.5%	100 μL for 20 mL	−20°C
dbCAMP (100 mM)	100 μm	20 μL for 20 mL	−20°C
DAPT (10 mM)	10 μM	20 μL for 20 mL	−20°C

Store at 4°C for up to 1 month.

**Table:** Assembloid Growth Medium (AGM)

Reagent	Final concentration	Amount	Storage
BrainPhys	N/A	224 mL	4°C
ES FBS ∗Do not filter∗	5%	12.5 mL	−20°C
N2 Supplement ∗Do not filter∗ (100×)	1×	2.5 mL	−20°C
B27 without vitamin A ∗Do not filter∗ (50×)	1×	5 mL	−20°C
GlutaMAX (100×)	1×	2.5 mL	22°C
Non-Essential Amino Acids (100×)	1×	2.5 mL	4°C
Ascorbic acid	200 μM	1 mL	4°C
**Total**	**N/A**	**250 mL**	**4°C**
BDNF (10 μg/mL)	20 ng/mL	40 μL for 20 mL	−80°C
GDNF (10 μg/mL)	20 ng/mL	40 μL for 20 mL	−80°C
Thiazovivin (4 mM)	2 μM	10 μL for 20 mL	−20°C
DAPT (10 mM)	10 μM	20 μL for 20 mL	−20°C
dbCAMP (100 mM)	100 μM	20 μL for 20 mL	−20°C

Store at 4°C for up to 1 month.

**Table:** Artificial Cerebrospinal Fluid (ACSF)

Reagent	Final concentration	Amount	Storage
NaCl Stock (1250 mM)	125 mM	100 mL	22°C
KCl, NaHCO_3_, NaHPO_4_ stock buffer (25, 260, 12.5 mM)	2.5, 26, 1.25 mM	100 mL	22°C
CaCl_2_ (1 M)	2 mM	2 mL	22°C
MgCl_2_ (1 M)	2 mM	2 mL	22°C
Glucose (1 M)	20 mM	20 mL	4°C
**Deionized water**		**To 1 L**	**22°C**

Adjust to 300–310 mOsm. Equilibrate with 95% O_2_/5% CO_2_. Prepare fresh. If preparing the day before, store at 4°C.

**Table:** Cesium Methanesulfonate Internal Pipette Solution

Reagent	Final concentration	Amount	Storage
CsMeSO_3_	125 mM	1.43 g	22°C
CsCl	2 mM	16.84 mg	22°C
HEPES	10 mM	119.15 mg	22°C
EGTA	0.1 mM	1.9 mg	22°C
ATP-Mg_2_	4 mM	101.44 mg	−20°C
GTP-Na	0.3 mM	7.85 mg	−20°C
Phosphocreatine-Na_2_	10 mM	127.5 mg	−20°C
QX-314 chloride	5 mM	74.7 mg	22°C
TEA-Cl	5 mM	41.43 mg	22°C
**Deionized water**		**To 50 mL**	**Store aliquots at −80°C**

Adjust pH to 7.4 and osmolarity to 290–295 mOsm.

**Table:** 1% Hydrochloric acid

Reagent	Final concentration	Amount	Storage
HCl (1 N)	1%	10 mL	22°C
Deionized water	N/A	Up to 1 L	22°C

***Note:*** This protocol has been developed for reporter hiPSC lines grown in culture under feeder-free conditions on 6-well tissue culture plates coated with hES-qualified Matrigel diluted 1:125. The protocol can be applied to hiPSC lines grown in culture on feeders with some modifications of the early differentiation steps.

***Alternatives:*** The ROCK inhibitor Y-27632 can be used instead of Thiazovivin.

## Step-by-step method details

### Differentiating hiPSC line into thalamic organoids

**Timing: 90 days**

**Figure 1:**
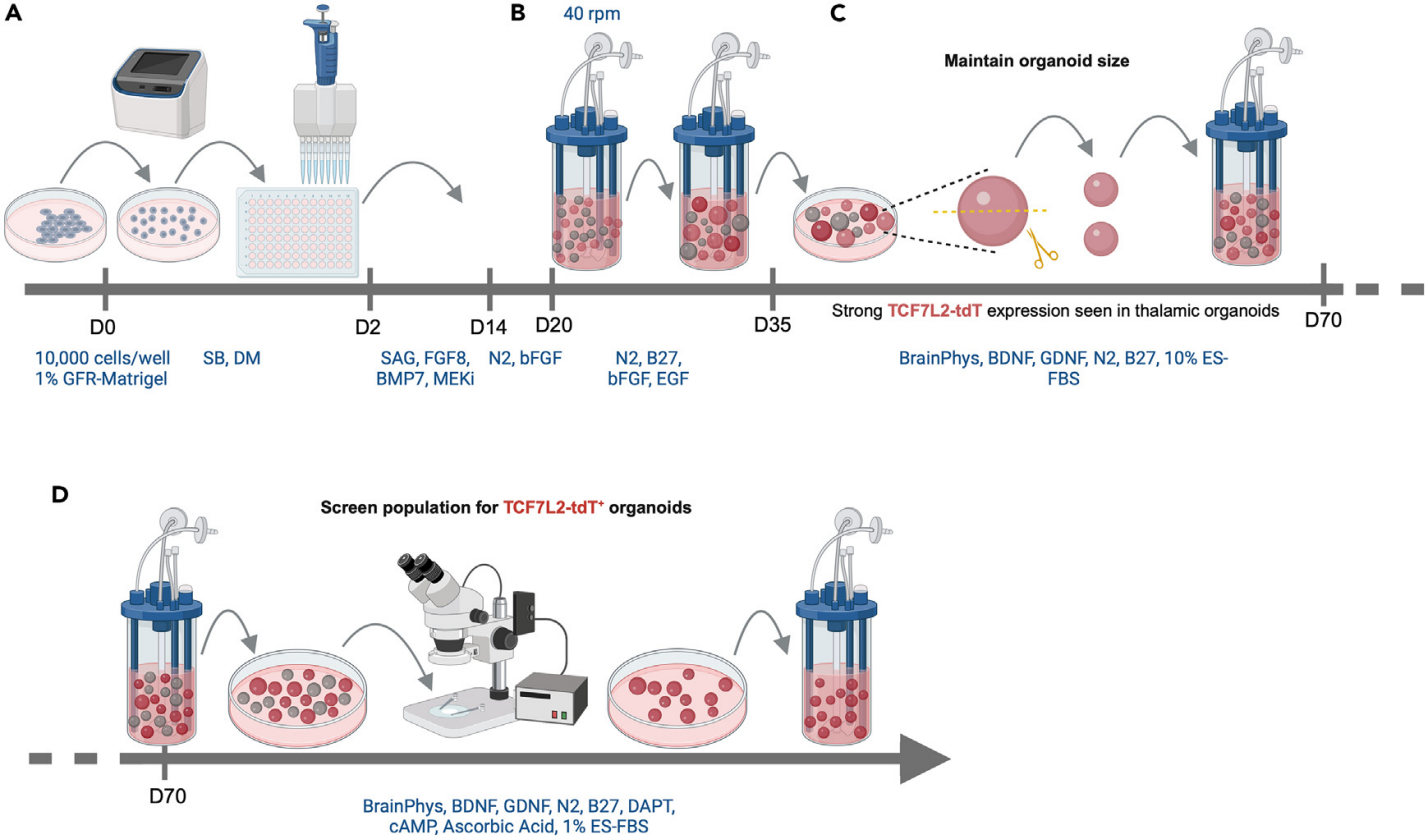
Schematic of the protocol used for differentiating hiPSCs into thalamic organoids (A) Plating of 10,000 cells/well to initiate thalamic lineage specification and EB formation. (B) Transfer of organoids to a bioreactor to facilitate nutrient and gas exchange. (C) Maintenance of organoid size by regularly cutting organoids in half. (D) Screening of the organoid population to select those with strong TCF7L2-tdT fluorescence.

This step of the protocol covers the directed differentiation of the thalamic reporter hiPSC line TP-190a-TCF7L2-tdT into thalamic organoids that will subsequently be fused with cortical organoids. This includes the procedures to generate embryoid bodies from hiPSCs, to induce differentiation towards the thalamic lineage, and to grow them into mature thalamic organoids (Figure 1).

1. Detach and dissociate hiPSCs (Figure 1A).
  a. Start differentiation when the hiPSCs are at approximately 70%–80% confluency.
  b. On D0, aspirate the medium from the 6-well plate and add 1 mL of Accutase, thawed at 22°C, to each well.
  c. Leave the plate at 37°C for 3–4 min.
  d. Remove the plate and tap it gently on the side to detach all loosely attached cells from the bottom of the well.
  e. Add 3 mL of mTeSR1 medium supplemented with 2 μM Thiazovivin (warmed to 22°C) to each well of the Accutase cell suspension.
    i. Pipette the contents up and down 8–10 times using a regular P1000 tip, to obtain a single-cell suspension.
  f. Transfer the entire cell suspension to a 15-mL tube and centrifuge it at 300 × *g* for 4 min.
  g. Aspirate almost all of the supernatant but leave a very small volume behind.
  h. Tap the cell pellet to obtain a dense, homogeneous cell suspension.
  i. Depending on the size of the pellet, add 1–2 mL of mTeSR1 supplemented with 2 μM Thiazovivin to the pellet.
  j. Tap to obtain a homogeneous cell suspension.
  k. Count the cells on an automated cell counter, according to the manufacturer’s instructions (https://logosbio.com/luna-ll/).
  l. Transfer a volume of this suspension containing 1 × 10^6^ cells to another 15-mL tube and centrifuge it at 300 × g for 4 min.
    ***Note:*** To plate 10,000 cells per well in a V-bottom low-attachment 96-well plate (96V plate), you will need 960,000 cells. To account for potential pipetting errors, calculate the cell number and the subsequent thalamic EB medium (TEM) volume required for a total of 100 wells.
  m. Aspirate almost all of the supernatant but leave a very small volume behind.
  n. Tap the cell pellet to obtain a homogeneous cell suspension.
2. Plate hiPSCs in a 96V plate.
  a. Add 10 mL of TEM supplemented with 1% v/v growth factor reduced Matrigel (GFR-Matrigel) to the 1 × 10^6^ cell suspension.
    i. Pipette the mixture up and down using a serological pipette, to obtain a homogeneous cell suspension.
    ii. Transfer it to a reservoir.
  b. Using a multichannel pipette, transfer 100 μL (10,000 cells) of cell suspension from the reservoir to each well of a low-attachment 96V plate.
  c. Centrifuge the plate at 100 × g for 2 min.
    i. Turn the plate through 180° and centrifuge it again at 100 × g for 2 min.
  d. Leave the plate in a normoxic incubator at 37°C, 5% CO_2_, and 20% O_2_.
3. Induce specification of thalamic lineage.
  a. On D2, replace 50 μL/well with TEM.
  b. On D4 and D6, replace 50 μL/well with TSM.
  c. From D8 to D12, replace 75 μL/well with TNIM every 2 days.
  d. From D14 to D18, replace 50 μL/well with TNDM I every 2 days.

**Figure 2:**
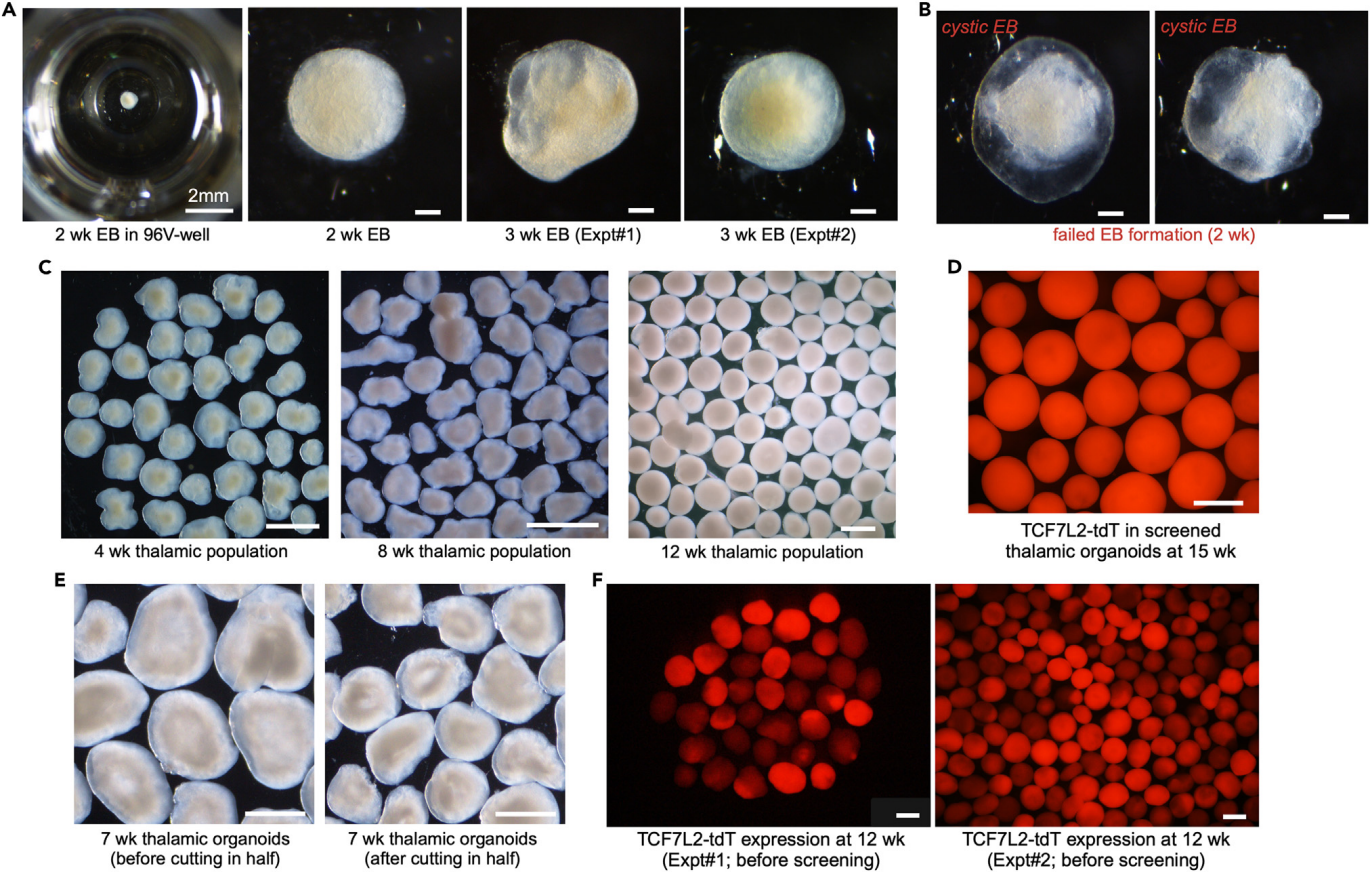
Morphology of thalamic organoids during development (A) Bright-field images of thalamic EBs at 2 weeks and 3 weeks after the start of differentiation. The images show the variability in EB morphology between two experiments (Expt#1 vs. Expt#2). Scale bar: 200 μm. (B) Bright-field images depicting failed EB formation at 2 weeks after the start of differentiation. Scale bar: 200 μm. (C) Representative bright-field images of a thalamic organoid population at 4 weeks, 8 weeks, and 12 weeks from the start of differentiation. The images show the smooth, translucent edges that appear after 4 weeks of development and the disproportionate horizontal expansion of thalamic organoids by 8 weeks. Scale bar: 1000 μm. (D) Strong TCF7L2-tdT fluorescence in 15-week organoids after screening; these can now be used to generate assembloids. Scale bar: 1000 μm. (E) Bright-field images of 7-week thalamic organoids before and after being cut in half. Scale bar: 1000 μm. (F) TCF7L2-tdT fluorescence in a population of 12-week organoids in two independent differentiation experiments before screening. The images show the variability in the level of TCF7L2-tdT induction, the yield of TCF7L2-tdT^+^ organoids, and the distribution of organoid sizes. Scale bar: 1000 μm.

***Note:*** By 2 weeks after plating, embryoid bodies (EBs) are expected to grow to a size of 800–900 μm in diameter. The morphology of the EBs may differ between independent experiments (Figure 2A). If the EBs appear cystic at 2 weeks, this indicates that the differentiation has failed, and the experiment should be restarted (Figure 2B). The efficiency of the thalamic lineage specification, the overall yield, and the size distribution of the organoids can vary between repeat differentiations of the same hiPSC line (Figure 2F).

4. Induce thalamic differentiation (Figure 1B).
  a. On D20, transfer all of the EBs from a 96V plate into two ABLE Biott disposable bioreactor units in 15 mL of TNDM II each.
    i. Place these on the ABLE Biott magnetic stir unit.
    ii. Set to 40 rpm and continue growing under these conditions henceforth.
  b. On D22 and D24, replace half of the medium with TNDM III supplemented with 20 ng/mL bFGF and 20 ng/mL EGF.
  c. On D26 and D28, replace half of the medium with TNDM III supplemented with 10 ng/mL bFGF and 10 ng/mL EGF.
  d. On D30 and D32, replace the entire medium in each bioreactor with TNDM III.
  e. Starting on D35, replace the entire medium with TNMM I every 4 days.
  f. Monitor the size of the organoids starting on D40.
    i. Cut them in half using the clipper scissors (Fine Science Tools, #15300-00) under a stereomicroscope when they grow to more than approximately 1200 μm in diameter (Figures 1C and 2E). See Video S1.
    ii. It may be useful to add 2 μM Thiazovivin to the bioreactor after cutting the organoids in half to mitigate the level of cell death.
    iii. Repeat the process approximately once a week until D90.

***Note:*** As an alternative to the ABLE Biott magnetic stir and disposable bioreactor system, organoids can be grown on an orbital shaker in low-attachment tissue culture plates with constant agitation at 90–110 rpm. Growing organoids with continuous agitation facilitates nutrient and gas exchange and waste diffusion. This helps to reduce the level of necrosis in the inner core of the organoid that is caused by the inability to access nutrients in the medium.^5^^,^^17^ In our experience, 9-week-old cortical organoids grown without agitation are significantly smaller than those grown with agitation, they have a proportionally larger necrotic core, and they show higher expression of genes associated with hypoxia. Other laboratories have reported dramatic improvements in cell survival^51^ and low inter-individual variability^52^ among organoids grown with constant agitation.

***Note:*** The purpose of cutting organoids in half regularly is to control the size of the necrotic core. The formation of this necrotic core is inevitable because of the lack of access to oxygen and nutrients (see above). When organoids are halved, the cellular layers previously shielded from the medium are exposed to it, thereby helping to mitigate cell death in these layers (Figures 1C and 2E). We recommend measuring the size of the organoids once a week, and when this size exceeds ∼2000 μm–2500 μm, they should preferably be cut in half.

***Note:*** Between D35 and D60, thalamic organoids often increase more in length (up to 3000 μm) than in width, resulting in organoids that appear “flat” and thick (Figure 2C). These organoids should be cut to sizes smaller than 1200 μm. Within a population, organoid sizes can range from 500 μm to 1200 μm in diameter.

**CRITICAL:** When organoids are left to grow unchecked, they tend to grow larger until they eventually disintegrate into pieces that appear unhealthy, with clear signs of cell death. To maintain healthy organoids in culture, it is critical to keep their size between 500 μm and 1200 μm in diameter by cutting them in half regularly, usually once every week (Figures 2C and 2D). We have observed that when the size of organoids is maintained within a range of 500 μm–1200 μm in diameter, the necrotic cores are smaller and the expression of genes associated with hypoxia is low. In addition, organoids have less tendency to disintegrate or ‘burst’ into pieces^53^ during long-term culture in the bioreactor.

5. Grow thalamic organoids to maturation.
  a. Starting on D70, replace the entire medium with TNMMII every 4 days.
6. Between D65 and D85, screen the organoid population for TCF7L2-tdT^+^ organoids (Figure 1A).
  a. Transfer the organoids from the bioreactor to a 6-well plate.
  b. Under the Discovery.V12 check the tdTomato fluorescence (indicative of TCF7L2 expression level) in the population.
  c. Select organoids with strong tdTomato fluorescence (whether patchy or whole-organoid) for continued maintenance in culture in a bioreactor (Figure 2D).
  d. Discard organoids with weak or moderate tdTomato fluorescence.

***Note:*** During screening, use sterile P10 tips to sift through the population and a wide-bore P1000 tip to handle the selected “good” organoids.

***Note:*** Organoids within a population often vary in size (Figure 2C). This is normal and will not affect the outcome of the experiment.

**CRITICAL:** Because of the experimental variability (often seen in hiPSC-based organoid model systems)^5^^,^^37^ with the current version of this protocol, the efficiency of thalamic lineage specification in the same hiPSC line can vary markedly between repeat differentiations (Figure 2F). Accordingly, it is imperative to use a reporter line.

Video S1. Cutting organoids in half with clipper scissors, related to steps 4(f) and 10(f)

### Differentiating hiPSC line into cortical organoids

**Timing: 90 days**

**Figure 3:**
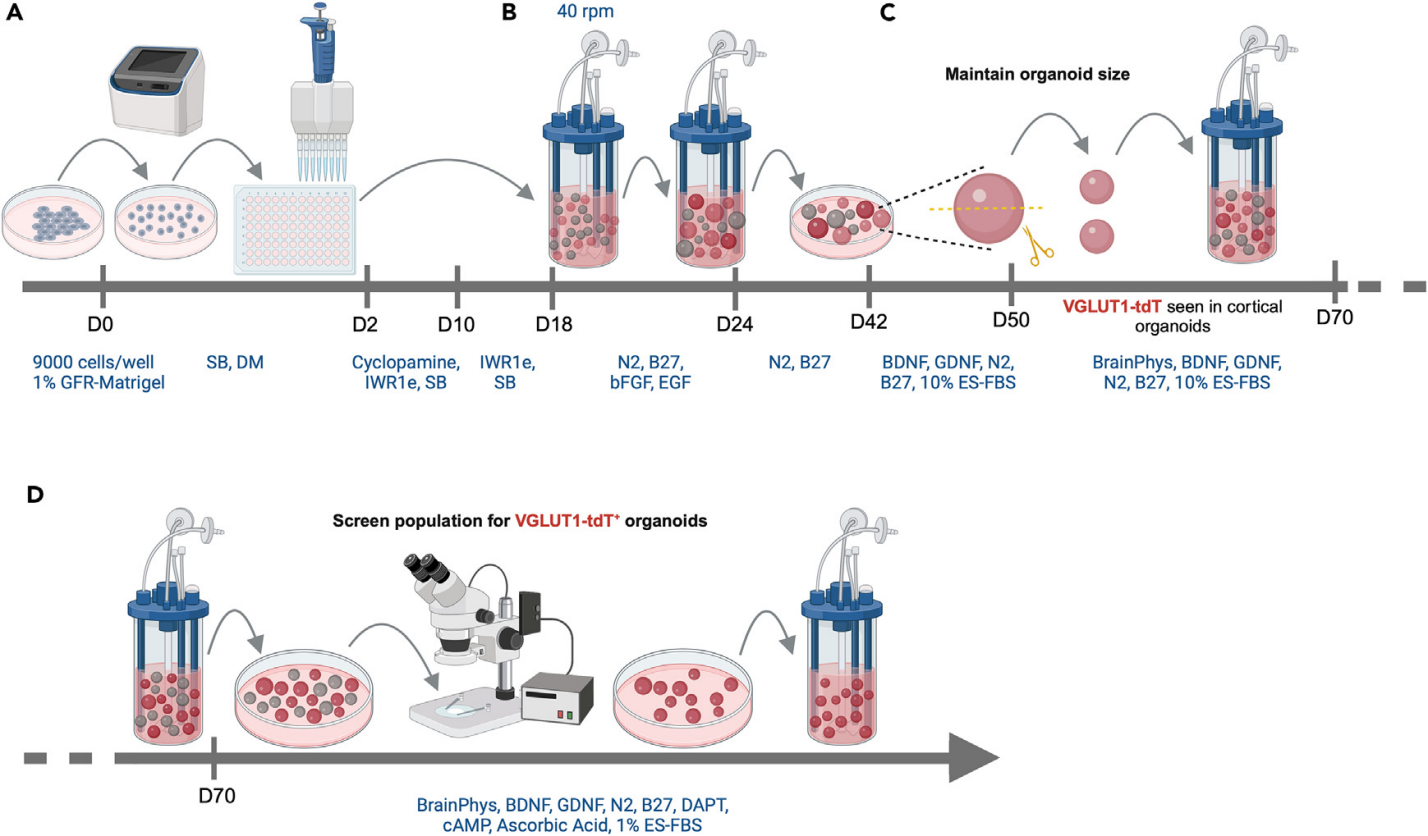
Schematic of the protocol used for differentiating hiPSCs into cortical organoids (A) Plating of 9000 cells/well to initiate thalamic lineage specification and EB formation. (B) Transfer of organoids to a bioreactor to facilitate nutrient and gas exchange. (C) Maintenance of organoid size by regularly cutting them in half. (D) Screening of the organoid population to select VGLUT1-tdT^+^ organoids.

This step of the protocol covers the directed differentiation of the cortical reporter hiPSC line TP-190a-VGLUT1-tdT into cortical organoids that will subsequently be fused with thalamic organoids. This includes the procedures to generate EBs from hiPSCs, to induce differentiation towards the cortical lineage, and to grow them into mature cortical organoids (Figure 3).

7. Detach and dissociate hiPSCs (Figure 3A).
  a. Start differentiation when the hiPSCs are at approximately 70%–80% confluency.
  b. On D0, aspirate the medium from the 6-well plate and add 1 mL of Accutase, thawed at 22°C, to each well.
  c. Leave the plate at 37°C for 3–4 min.
  d. Remove the plate and tap it gently on the side to detach all loosely attached cells from the bottom of the well.
  e. Add 3 mL of mTeSR1 medium supplemented with 2 μM Thiazovivin (warmed to 22°C) to each well of the Accutase cell suspension.
    i. Pipette the mixture up and down 8–10 times using a regular P1000 tip, to obtain a single-cell suspension.
  f. Transfer all of the cell suspension to a 15-mL tube and centrifuge it at 300 × *g* for 4 min.
  g. Aspirate almost all of the supernatant but leave a very small volume behind.
  h. Tap the cell pellet to obtain a dense, homogeneous cell suspension.
  i. Depending on the size of the pellet, add 1–2 mL of mTeSR1 supplemented with 2 μM Thiazovivin.
    i. Tap to obtain a homogeneous cell suspension.
  j. Count the cells on an automated cell counter, according to the manufacturer’s instructions (https://logosbio.com/luna-ll/).
  k. Transfer a volume of this suspension containing 0.9 × 10^6^ cells to another 15-mL tube.
  l. Centrifuge at 300 × *g* for 4 min.
    ***Note:*** To plate 9000 cells per well in a 96V plate, you will need 864,000 cells. To account for potential pipetting errors, calculate the cell number and the subsequent CEM volume required for a total of 100 wells.
  m. Aspirate almost all of the supernatant but leave a very small volume behind.
  n. Tap the cell pellet to obtain a homogeneous cell suspension.
8. Plate hiPSCs in a 96V plate (Figure 3A).
  a. Add 10 mL of CEM supplemented with 1% v/v GFR-Matrigel to the 0.9 × 10^6^ cell suspension.
    i. Pipette the mixture up and down using a serological pipette, to obtain a homogeneous cell suspension.
    ii. Transfer it to a reservoir.
      ***Note:*** In our experience, adding Matrigel to the D0 media improves the efficiency of EB formation and the appearance of EB’s during the first week.
  b. Using a multichannel pipette, transfer 100 μL of cell suspension (= 9000 cells) from the reservoir to each well of a 96V plate.
  c. Centrifuge the plate at 100 × *g* for 2 min.
    i. Turn the plate 180°.
    ii. Centrifuge again at 100 × *g* for 2 min.
  d. Leave the plate at 37°C, 5% CO_2_, and 20% O_2_.
9. Induce specification of cortical lineage (Figure 3A).
  a. On D2, replace 50 μL/well with CEM.
  b. From D4 to D8, replace 50 μL/well with CSM every 2 days.
  c. From D10 to D16, replace 50 μL/well with CNIM every 2 days.

**Figure 4:**
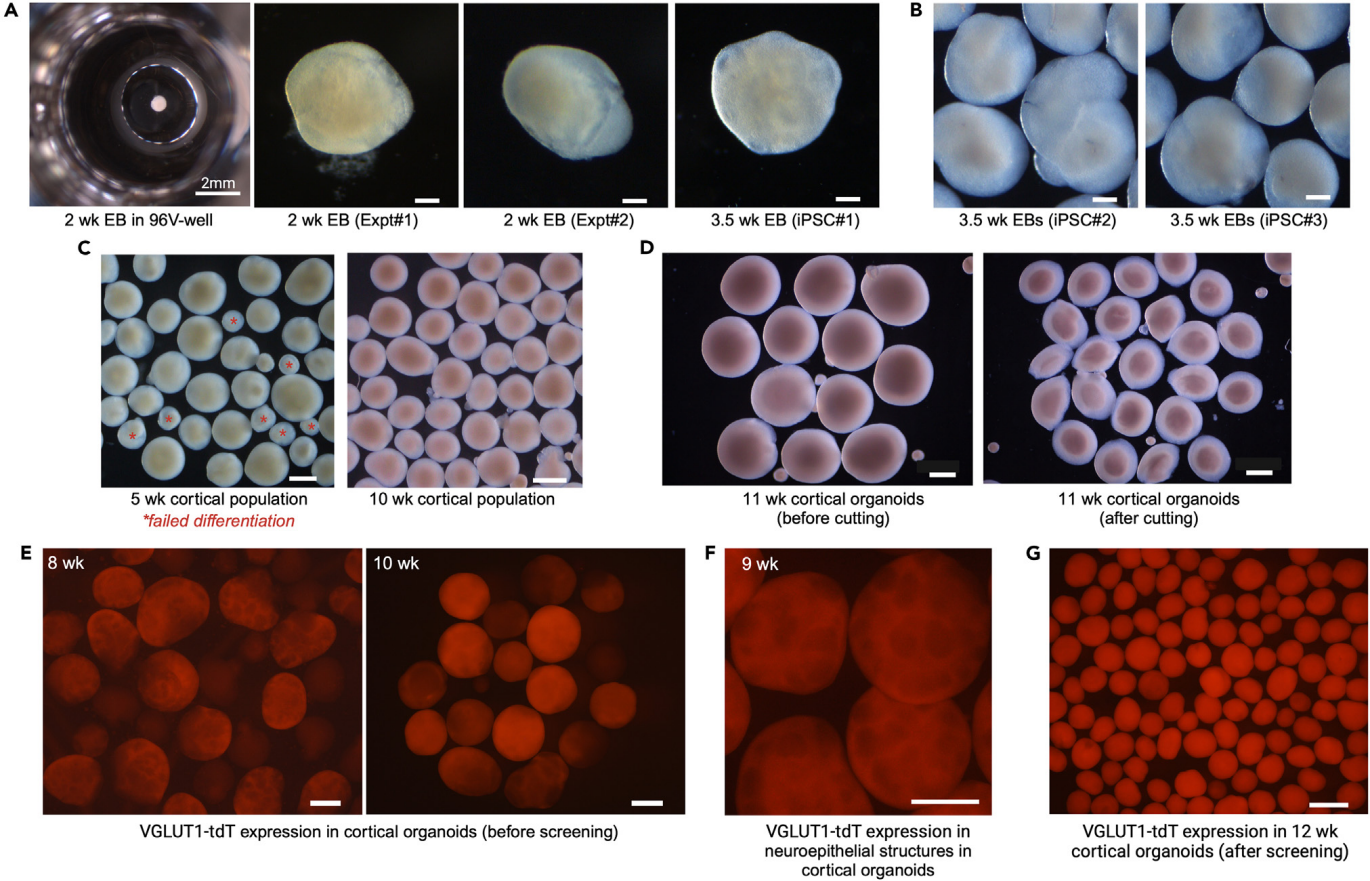
Morphology of cortical organoids during development (A) Bright-field images of cortical EBs at 2 weeks and 3.5 weeks after the start of differentiation. The images show the variability in 2-week EB morphology between experiments (Expt#1 vs. Expt#2) and between three hiPSC lines at 3.5 weeks (iPSC#1 vs. iPSC#2 vs. iPSC#3). Scale bar: 200 μm. (B) Bright-field images of 3.5-week cortical EBs differentiated from two different hiPSC lines, using the same protocol. Scale bar: 200 μm. (C) Representative bright-field images of a cortical organoid population at 5 weeks and 10 weeks after the start of differentiation. Scale bar: 500 μm. (D) Bright-field images of 11-week cortical organoids before and after being cut in half. Scale bar: 500 μm. (E) VGLUT1-tdT fluorescence in two independent populations of 8-week and 10-week cortical organoids before screening. The images show the variability in the level of VGLUT1-tdT induction, the yield of VGLUT1-tdT^+^ organoids, and the distribution of their sizes. Note the VGLUT1-tdT^+^ neuroepithelial structures at 8 weeks. Scale bar: 500 μm. (F) Fluorescence image of 9-week VGLUT1-tdTomato^+^ cortical organoids with multiple neuroepithelial structures in each. Scale bar: 500μm. (G) VGLUT1-tdT fluorescence in 12-week cortical organoids after screening. Scale bar: 1000 μm.

***Note:*** By 2 weeks after plating, EBs are expected to grow to a size of 800–900 μm. EBs may differ in size and shape between independent experiments (Figure 4A) and they are not always spherical, but they will continue to grow into similar looking organoids by 4–5 weeks. Note that, because we routinely cut organoids to maintain their size, older organoids are denser than younger EBs but not much larger (Figure 4C).

***Note:*** Some hiPSC lines respond poorly to the differentiation protocol, but among those that have strong neurogenic differentiation potential, the organoid morphology is quite similar when subjected to the same protocol (Figure 4B).

***Note:*** In some differentiations, a fraction of EBs fail to develop beyond the first couple of weeks, resulting in small spherical cell clusters in which all cells appear to be dying (Figure 4B).

10. Induce cortical differentiation (Figure 3B).
  a. On D18 and D20, replace 50 μL/well with CNDM I.
  b. On D22, transfer all EBs from a 96V plate into two bioreactor units in 15 mL of CNDM I each.
    i. Place them on the magnetic stir unit.
    ii. Set to 40 rpm.
  c. From D24 to D28, replace half of the medium in the bioreactor with CNDM II every 2 days.
  d. From D30 to D38, replace the entire medium with CNDM III every 4 days.
  e. On D42 and D46, replace the entire medium with CNMM I every 4 days.
  f. Monitor the size of the organoids starting on D40.
    i. Cut them in half using fine scissors under a stereomicroscope when they grow to more than approximately 1200 μm in diameter (Figures 3C and 4D). See Video S1.
    ii. It may be useful to add 2 μM Thiazovivin to the bioreactor after cutting the organoids in half to mitigate the level of cell death.
    iii. Repeat approximately once a week until D90, after which the organoids do not grow much.

***Note:*** As an alternative to the ABLE Biott magnetic stir and disposable bioreactor system, organoids can be grown on an orbital shaker in low-attachment tissue culture plates with constant agitation at 90–110 rpm (see the “Notes” following step 4).

***Note:*** The purpose of cutting organoids in half regularly is to control the size of the necrotic core. The formation of this necrotic core is inevitable because of the lack of access to oxygen and nutrients. When organoids are halved, the cellular layers previously shielded from the medium are exposed to it, thereby helping to mitigate cell death in these layers (Figures 3C and 4D). We recommend measuring the size of the organoids once a week, and when this size exceeds ∼2000 μm–2500 μm, they should preferably be cut in half.

**CRITICAL:** Allowing organoids to grow to more than 1200 μm often leads to them disintegrating. This is accompanied by massive amounts of cell death, so that the organoids are no longer salvageable. When the organoids are maintained within a size range of 500 μm–1200 μm in diameter, we have empirical evidence that the necrotic cores are smaller.

11. Grow cortical organoids to maturation.
  a. From D50 to D66, replace the entire medium with CNMM II every 4 days.
  b. Starting on D70, replace the entire medium with CNMM III every 4 days.
12. Between D80 and D90, screen the organoid population for VGLUT1-tdT^+^ organoids (Figure 3D).
  a. Transfer the organoids from the bioreactor to a 6-well plate.
  b. Under the Discovery.V12, check the VGLUT1-tdT fluorescence in the population.
  c. Select VGLUT1-tdT^+^ organoids for continued maintenance in culture in a bioreactor; discard the rest.

***Note:*** VGLUT1-tdT fluorescence in cortical organoids can be detected as early as 6 weeks in a pattern of multiple neuroepithelial structures that continue to be seen until at least 9 weeks (Figures 4E and 4F). Over time, the level of VGLUT1-tdT fluorescence in cortical organoids increases while the neuroepithelial structures disappear and the signal becomes more uniform throughout the organoid (Figure 4G). Because this protocol can be less than 100% efficient, populations of organoids before screening often comprise organoids with VGLUT1-tdT fluorescence levels ranging from strong to absent (Figure 4E).

***Note:*** During screening, use sterile P10 tips to sift through the population and a wide-bore P1000 tip to handle the selected “good” organoids.

**CRITICAL:** Because of the experimental variability (often seen in hiPSC-based organoid model systems) with the current version of this protocol, the efficiency of cortical lineage specification in the same hiPSC line can vary markedly between repeat differentiations (Figure 4E). Accordingly, it is imperative to use a reporter line.

### Generating and maintaining thalamocortical assembloids

**Timing: 30–60 days**

**Figure 5:**
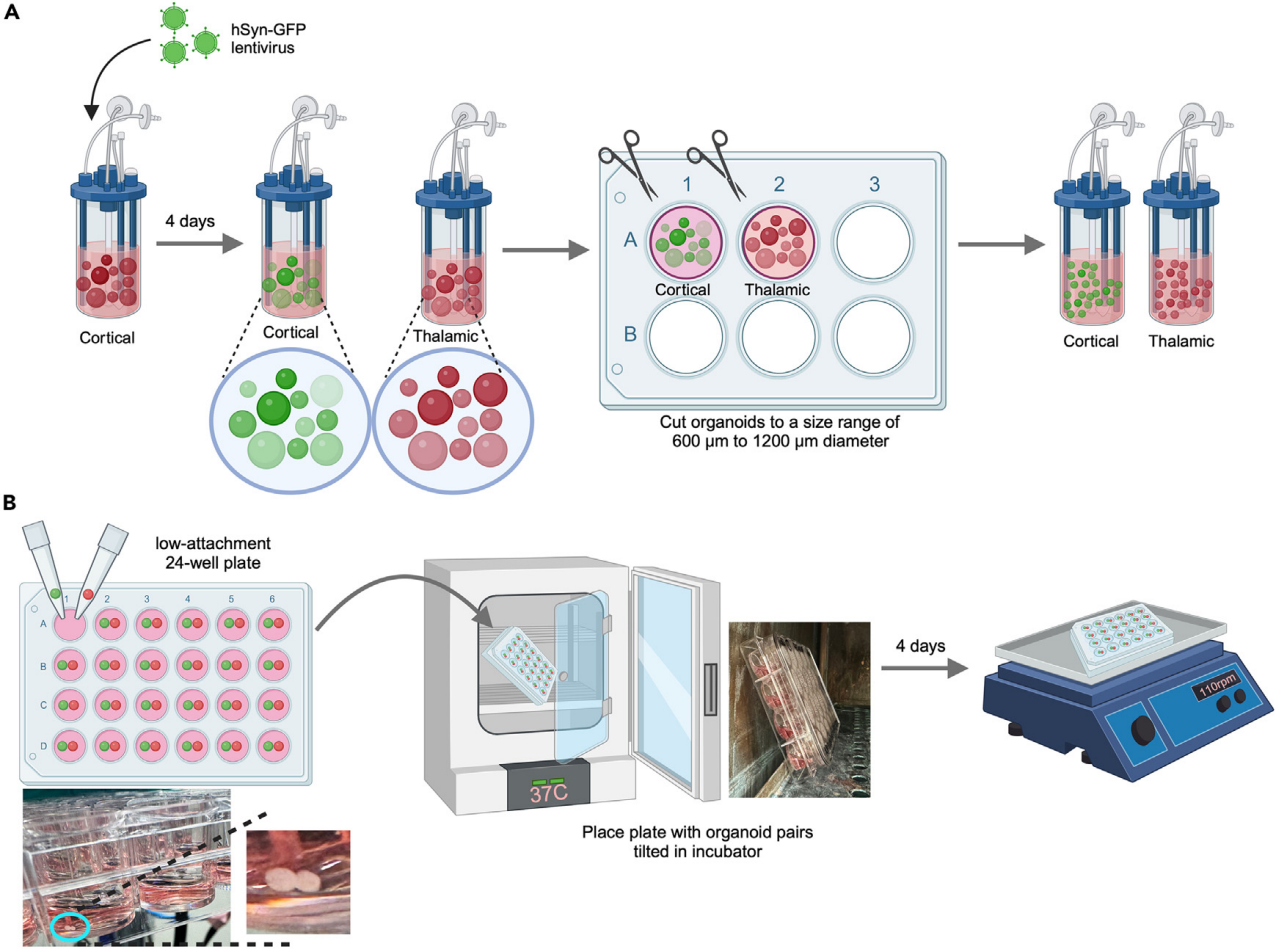
Schematic of the steps involved in generating thalamocortical assembloids (A) Preparation of cortical and thalamic organoids for fusion. (B) Plating organoids for generating and growing thalamocortical assembloids.

This step of the protocol covers the procedures to create, monitor, screen, and grow assembloids from D90 and older VGLUT1-tdT^+^ cortical and TCF7L2-tdT^+^ thalamic organoids (Figure 5).

13. Transduce cortical organoids with hSyn-GFP lentivirus (Figure 5A).
  a. At 3–7 days before the start of fusion, thaw on ice one aliquot of the hSyn-GFP lentivirus stored at −80°C.
  b. Remove approximately 10 mL of spent medium from the bioreactor containing the cortical organoids, leaving approximately 10 mL of medium.
  c. Calculate the amount of hSyn-GFP lentivirus that will be needed for 10 mL of medium and a final concentration of approximately 50 × 10^6^ TU/mL (a 1:200 dilution of a 10^9^ TU/mL stock), and the amount of Polybrene that will be needed for a final concentration of 2 μg/mL.
    ***Note:*** The lentivirus was generated by the Vector Core at St Jude using standard procedure. In our experience, the efficiency of lentiviral transduction in organoids is markedly higher in the presence of Polybrene, which is a cationic polymer with coagulant properties.
  d. Remove 2 mL from the 10 mL of old medium in the bioreactor.
  e. Add the amounts of hSyn-GFP and Polybrene calculated above.
    i. Mix well.
    ii. For example, add 50 μL of hSyn-GFP stock at 10^9^ TU/mL and 2 μL of a 10 mg/mL Polybrene stock to 2 mL of spent medium.
  f. Add this lentivirus–Polybrene mix dropwise to the bioreactor containing all of the cortical organoids and the remaining 8 mL of spent medium.
  g. Leave the bioreactor on the magnetic stir unit at 40 rpm for 16–18 h.
  h. Next day (after at least 18 h), aspirate the lentivirus-containing medium from the transduced bioreactor and transfer it to bleach.
  i. Rinse the organoids in the bioreactor twice with 10 mL (per wash) of DMEM-F12.
  j. Add 20 mL of fresh medium to the bioreactor.
  k. Leave it on the magnetic stir unit at 40 rpm.
14. Prepare thalamic and cortical organoids for fusion (Figure 5A).
  a. At 3–4 days before the start of fusion, transfer the entire population of thalamic and cortical organoids from their respective bioreactors to one or two wells of a regular 6-well plate in approximately 4 mL of medium per well.
  b. Observe the TCF7L2-tdT fluorescence in the thalamic organoids under the Discovery.V12.
  c. Observe the VGLUT1-tdT and hSyn-GFP fluorescence in the cortical organoids under the Discovery.V12.
  d. Select thalamic organoids showing strong TCF7L2-tdT fluorescence throughout the organoid.
  e. Select cortical organoids showing strong VGLUT1-tdT and hSyn-GFP fluorescence.
    ***Note:*** In thalamic organoid cultures, organoids with weak, moderate, or patchy TCF7L2-tdT fluorescence can often be seen. It is best to discard these and not use them for fusion.
  f. Using sterilized fine scissors, cut selected organoids in half to obtain a population of TCF7L2-tdT^+^ and VGLUT1-tdT^+^ organoids that are 600 μm–1200 μm in diameter.
    **CRITICAL:** In our experience, to obtain the highest rates of fusion efficiency and healthy assembloid generation, both cortical and thalamic organoids should be no more than 1200 μm in diameter at the time of fusion. Larger organoids do not fuse efficiently and are more likely to undergo cell death and disintegration over time.
  g. Transfer these cut organoids to bioreactors in 20 mL of medium each.
  h. Leave them on the magnetic stir unit at 40 rpm.
15. Plate organoids for fusion into assembloids (Figure 5B).
  a. On D0, first transfer the organoids that were previously selected and cut to an appropriate size into one or two wells of a 6-well plate.
  b. Add 500 μL/well of OFM to a low-attachment 24-well plate (the fusion plate).
  c. Under the Discovery.V12 use a wide-bore P200 tip to pick and transfer one thalamic and one cortical organoid into each well of the fusion plate.
  d. After transferring organoids to all wells of the fusion plate, swirl the contents then slightly tilt the plate to allow the organoids to sink to the bottom of the wells.
  e. Using a sterile P10 tip, push the organoids to the very bottom of the wells in the tilted plate and towards each other so that they are as physically close as possible.
  f. Place the plate in the same tilted position at an angle of 45°–60° in the incubator, resting it along the side wall of the incubator.
  g. Leave the plate undisturbed for 4 days.

***Note:*** The organoid pairs in each well need not be of comparable size. As long as each organoid in the pair is between 500 μm and 1000 μm in diameter, the two organoids can be of dissimilar size. In our experience, plating organoids of dissimilar size does not affect the fusion efficiency or assembloid health.

16. Grow assembloids with agitation and regular medium changes (Figure 5B).
  a. On D4, remove the fusion plate from the incubator while keeping it tilted.
  b. Move the plate to the BSC and position it at a tilted angle to facilitate changing the medium.
  c. Remove 300 μL of old medium from each well.
    i. Position a P1000 tip at the side of the well and press it against the bottom of the well. Slowly pipette out the medium.
  d. Add 300 μL of fresh AGM to each well.
    i. Position a P1000 tip at the top of the well, press against the bottom, and slowly release the fresh medium into the well.
  e. Slowly tilt the plate back to a flat position and place it on an orbital shaker set at 50 rpm.
  f. Increase the speed of the orbital shaker to 60 rpm on D5.
    i. Increase to 70 rpm after approximately 6 h.
    ii. Increase to 80 rpm on D6.
    iii. Increase to 100 rpm on D7.
    iv. Increase to 110 rpm on D8.
    v. After D8, continue growing the assembloids on the orbital shaker at 110 rpm.
      ***Note:*** Most fused organoid pairs at this stage are loosely connected. Therefore, the medium should be replaced gently to minimize disturbance of the organoids.
  g. On D7 and D10, replace 300 μL of medium per well, as was previously done on D4.
  h. Starting on D13, replace as much medium as possible in each well.
    i. Use a vacuum line to gently aspirate as much medium as possible from each well.
    ii. Slowly pipette 500 μL of AGM into each well, using a serological pipette.
  i. Re-feed the organoids in the same way every 3–4 days until they are harvested for electrophysiological recording.
  j. Monitor the extent of fusion of the organoid pairs starting on D4 (Figure 6A).
    i. Discard organoid pairs that are still separated at D7.
    ii. Pairs of organoids that are only touching each other and for which the level of fusion does not appear to have increased between D4 and D28 can also be discarded on D28.
      ***Note:*** Different pairs of organoids fuse to different extents. The extent of fusion can be determined by the acuteness of the angle at the indentation or the point of fusion between the two organoids and by the amount of surface area of each organoid that is “fused” with the other organoid. For example, by D7, organoid fusion can range from “well fused” to “incompletely fused.” By D15, organoid fusion can range from “fully merged” to “well fused” to “incompletely fused” (Figure 6A).

**Figure 6:**
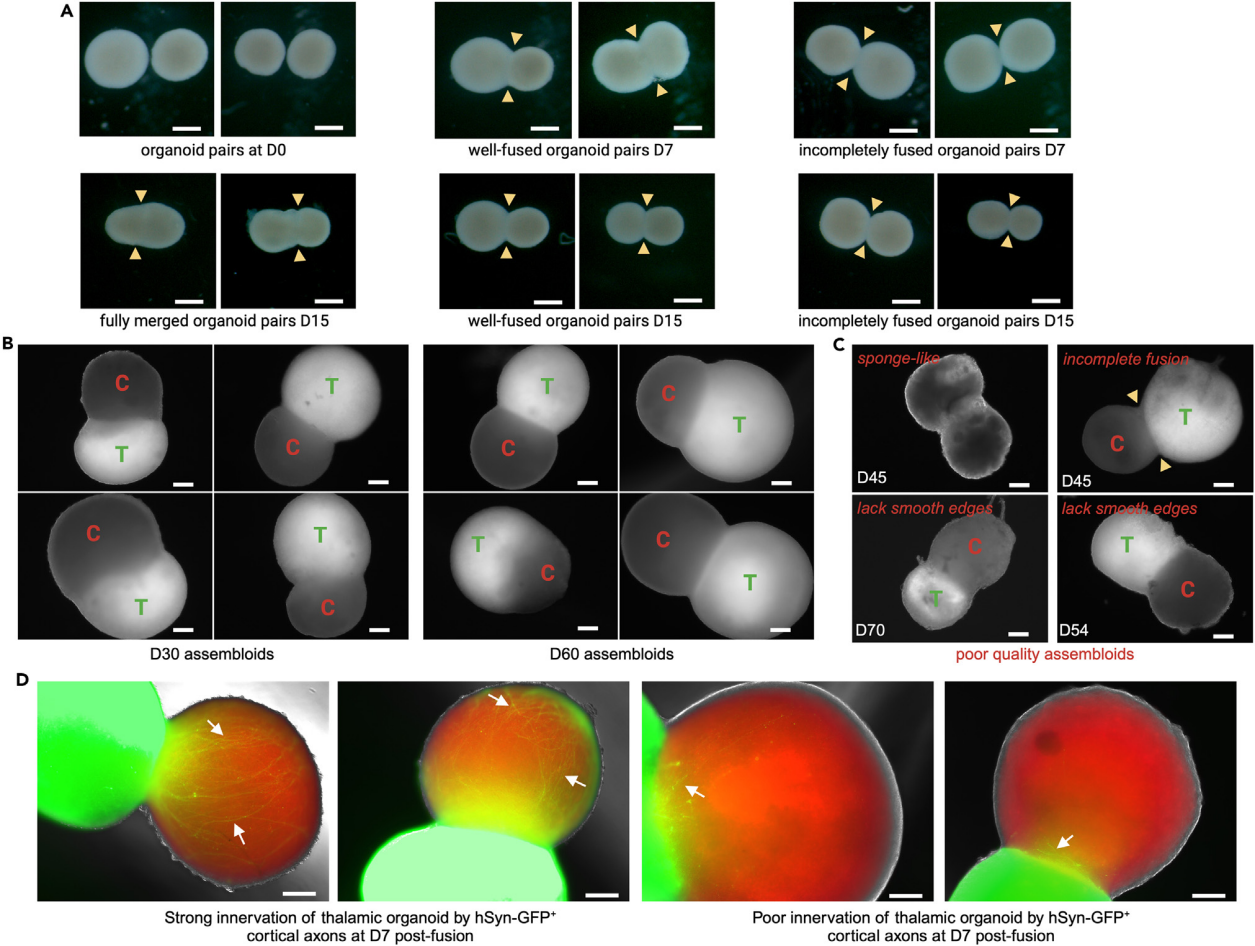
Morphology of thalamocortical organoids during development (A) Bright-field images of pairs of cortical and thalamic organoids plated for assembloid formation at D0 and their appearance at D7 and D15. The images show different levels of fusion, from fully merged/well fused to incompletely/partially fused. The level of fusion is indicated by the acuteness of the angle at the point of fusion between the two organoids (yellow arrowheads) and by the amount of surface area of each organoid that is “fused” with the other organoid. Scale bar: 500 μm. (B) Multiple assembloids generated in the same batch at D30 and D60 post-fusion. The images show various levels of fusion, the relative sizes of the cortical and thalamic halves, and the overall morphology of the assembloids. “C” indicates the cortical organoid and “T” indicates the thalamic organoid within the assembloid. Scale bar: 200 μm. (C) Examples of poor-quality assembloids (D45–D70) that have a “sponge-like” or “fluffy” appearance (a clear sign of cell death), are incompletely fused with each other (note the yellow arrowheads at the point of fusion), or lack smooth edges (indicating an unhealthy assembloid). Scale bar: 200 μm. (D) Fluorescence images of hSyn-GFP^+^ cortical axons innervating the TCF7L2-tdT^+^ thalamic organoid at D7 post-fusion. Note the variability in the extent of innervation, from strong to poor. Scale bar: 200 μm.
17. Starting on D30, select assembloids for electrophysiological recording.
  a. Under the Discovery.V12, observe the size and morphology of the assembloids in each well of the fusion plate.
  b. For best results, select assembloids in which the two halves appear to be fully merged or well fused, typically those for which the angle at the indentation between the two halves is more than 120° (Figure 6B).
  c. Our most successful recordings were obtained from assembloids that were between D30 and D70 at the time of recording.
    ***Note:*** Assembloids in which the two halves are not fully merged and in which the angle at the indentation is acute can also exhibit electrical responses as well as synaptic plasticity, albeit at a reduced rate. Therefore, depending on the overall yield of assembloids and the requirements of the experiment, these assembloids can also be used for electrophysiological recording.
    ***Note:*** Incompletely or partially fused assembloids, those that have a “sponge-like” or “fluffy” appearance (indicative of cell death), and those that lack smooth edges (indicative of an unhealthy assembloid) should not be used for electrophysiological analyses (Figure 6C).
  d. Under the Discovery.V12, observe the hSyn-GFP^+^ axons projecting from the cortical side into the thalamic side of the assembloid in each well.
  e. For best results, select assembloids with projections that appear to be dense and elaborate (Figure 6D).
    ***Note:*** Some fully merged and well-fused assembloids have dense hSyn-GFP^+^ cortical processes in the thalamic half at the time of harvesting, whereas in others these processes are sparse (Figure 6D). The latter assembloids can still be used successfully to establish electrophysiological recordings, but at a slightly lower rate than the former. Therefore, depending on the overall yield of assembloids and the requirements of the experiment, these assembloids can be used for electrophysiological experiments.

### Electrophysiological recording from thalamocortical assembloids

**Timing:Between 2 and 8 h(for steps 18–34)**

**Timing:Variable (Depending on the skill level of the electrophysiologist, it can take a few minutes to an hour to obtain a whole-cell recording.)(for steps 25–31)**

**Timing:Variable (Success in electrically evoking a synaptic response will be immediately apparent. If no response is detected, troubleshooting to verify this can take up to 30 min.)(for step 30)**

**Figure 7:**
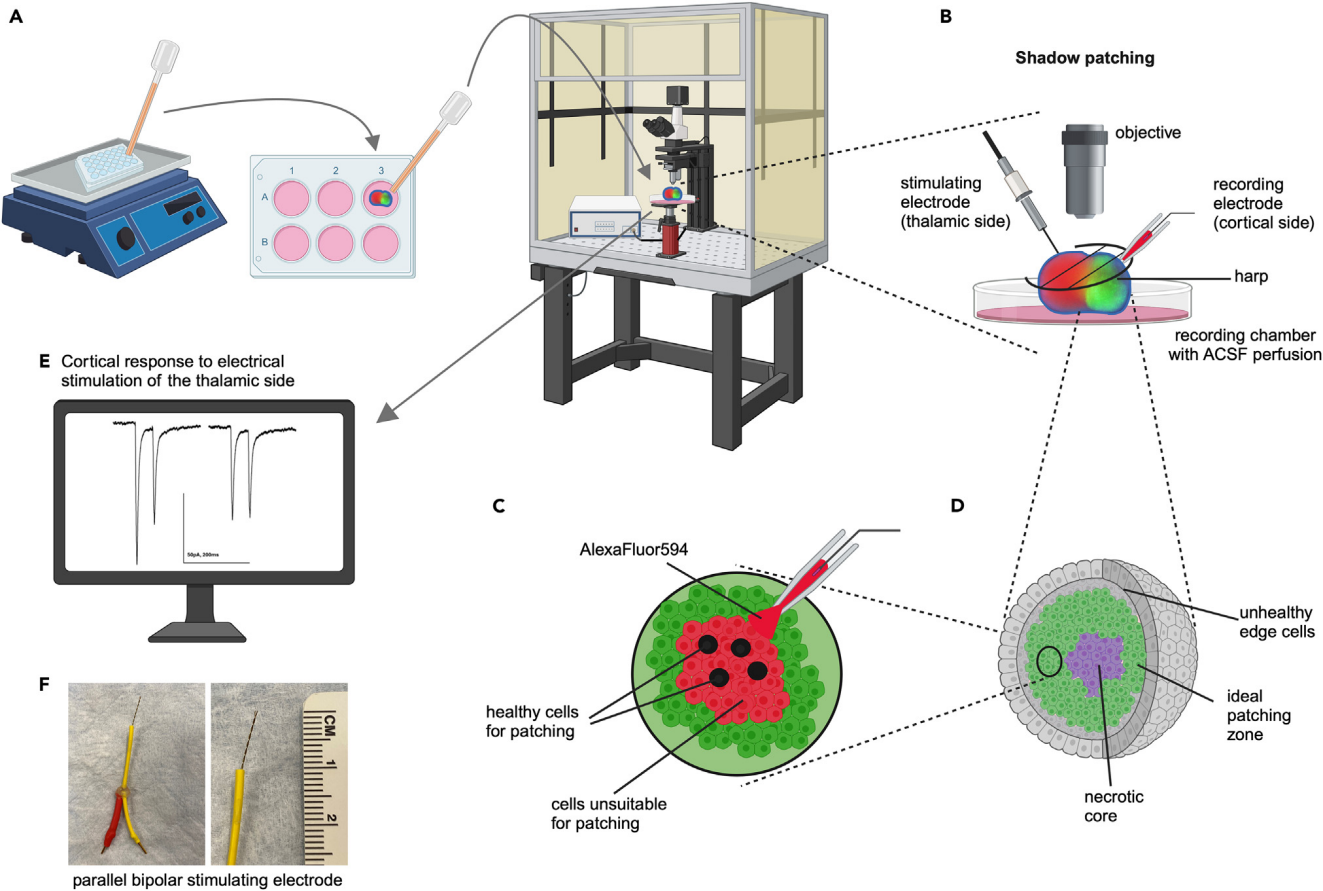
Schematic of the steps involved in whole-cell shadow patch-clamp recording from thalamocortical assembloids (A) Transferring the assembloid to the recording chamber of an electrophysiology rig. (B) The configuration for electrical stimulation and whole-cell patch-clamp recording. (C) Illustration of the basis of shadow patching. (D) Illustration of the quality of cells inside the assembloid and the ideal zone for patching. (E) Example traces of successful voltage-clamp recordings of synaptic transmission in the thalamocortical assembloid. (F) Example of a homemade parallel bipolar stimulating electrode, with magnification. Yellow and red outer layers are heat-shrink tubing. Male pins are soldered onto the ends of the two wires, which wrap around each other. The clear dot in the center is glue from a glue-gun added for stability.

This step of the protocol covers the procedures to prepare an assembloid for whole-cell patch-clamp recording and to perform shadow patching on the assembloid. We also detail an electrical stimulation protocol and the process by which to record synaptic transmission within the assembloid (Figure 7).

18. Approximately 1 h before recording, prepare standard artificial cerebrospinal fluid (ACSF) by using the recipe suggested or your preferred recipe.

***Note:*** Ideally, prepare fresh ACSF on the day of recording, but 1- to 2-day-old ACSF is also acceptable.

19. Oxygenate the ACSF with 95% O_2_ and 5% CO_2_. Store it at 22°C.
20. Prepare or thaw previously aliquoted internal pipette solution. This solution is prepared to mimic the intracellular contents of the recorded neuron.
  a. We suggest using cesium methanesulfonate (CsMeSO_3_) for voltage-clamp recordings. Please refer to the materials and equipment section for detailed composition.
  b. Keep the internal pipette solution on ice for the duration of the recording session.

***Note:*** We suggest preparing and aliquoting the internal pipette solution on a prior day. We recommend preparing aliquots of 400–500 μL and storing them at −80°C until use on the day of recording.

**CRITICAL:** To increase the visibility of healthy cells and to improve the chances of producing a gigaseal for successful patch-clamp recordings, we recommend using the technique of “shadow patching”^30^^,^^31^ (see step 27 for more details). For shadow patching, a fluorescent dye is added to the internal pipette solution and patching is performed under two-photon fluorescence imaging guidance. In this step, add 1 μL of Alexa Fluor 594 (or a dye that is compatible with your electrophysiology set-up) to 500 μL of internal pipette solution.

21. When both solutions are at 20°C–25°C, measure their osmolarities using a standard osmometer (see Equipment).
  a. Aim for a difference of 10–15 mOsm to aid in obtaining a gigaseal and to provide stability for long-term recordings.
22. Clean the electrophysiology rig perfusion system and recording chamber.
  a. Flush with approximately 100 mL of 1% hydrochloric acid (HCl).
  b. Next, flush with copious amounts (>250 mL) of deionized water.
  c. It is preferable to include the electrophysiology slice anchor and the stimulating electrode tip in the recording chamber during the cleaning process.

***Note:*** Follow the HCl perfusion immediately with deionized water perfusion. Do not let the HCl remain in the system for long or it will begin to corrode the tubing and recording chamber.

23. After the water flush, continuously perfuse oxygenated ACSF through the electrophysiology perfusion system.
  a. Use a chamber heater or inline heating system to warm the recording chamber ACSF to 32°C ± 1°C.
  b. Adjust the drip rate to approximately 2–3 mL/min.
  c. Allow the ACSF to perfuse for at least 5 min.
24. From the 24-well plate on the orbital shaker, select an assembloid with ideal morphology for recording (see step 17 and Figures 6B–6D).
25. Transfer the assembloid to the electrophysiology rig (Figure 7A).
  a. Transport the assembloid in one well of a 24-well plate, submerged in medium.
  b. Avoid excessive movement during transport.
  c. Bring the assembloid immediately to the electrophysiology rig with a delay of not more than 5 min.
  d. Transfer the assembloid to the recording chamber by using a transfer pipette with a widened tip (approximately 5–6 mm in diameter).

***Note:*** We cut the tip of a standard plastic transfer pipette to enable the large assembloid to pass easily into the pipette. If the transfer pipette is not widened, there is a risk of damaging the assembloid during the transfer process.

When drawing up the assembloid from the 24-well plate, you will also draw up some of the medium. Aim to transfer as little of the medium as possible to the recording chamber. The perfusion system will wash away excess medium.

**CRITICAL:** Take extra care that the assembloid does not come into close contact with the fluid perfusion vacuum or it will be aspirated and lost.

26. Position the assembloid under the microscope by nudging the edges with blunted forceps.
27. Place a standard electrophysiology slice anchor (or “harp”) over the assembloid, taking care not to sever the connections between the two sides.
  a. Place the harp in a way that leaves ample room on each side for electrode placement (Figure 7B).

**CRITICAL:** Ideally, to preserve tissue health, the harp should be placed once and not moved. Once the assembloid is in place in the chamber, it should not move. Tissue movement during long-term plasticity recordings will result in loss of cells and incomplete data collection.

28. Check for differences in hSyn-GFP fluorescence levels between the thalamic and cortical sides of the assembloid and, accordingly, determine the correct recording configuration.
29. To perform thalamocortical recordings, slowly lower the concentric bipolar stimulating electrode into the center of the thalamic side of the assembloid until the entire graded tip of the stimulation electrode is surrounded by tissue (Figure 7B).

***Note:*** Position the harp and the stimulating electrode so that the electrode does not press down on the harp. Such pressure can damage the underlying tissue and/or cause the assembloid to break apart.

30. Perform shadow patching on the cortical side of the assembloid (Figures 7B and 7C).
  a. Fill a borosilicate glass pipette with internal pipette solution (approximately 5 μL) and attach it to a headstage on a motorized micromanipulator.
  b. Lower the pipette into the ACSF and apply 0.2 mL of continuous positive pressure using a 1-mL syringe.
  c. Under high-magnification (20–60×) guidance, approach and enter the tissue by using the micromanipulator.
    i. The positive pressure through the pipette will cause the internal pipette solution containing Alexa Fluor 594 to be released into the tissue (Figure 7C).
    ii. Unhealthy and dead cells, as well as the surrounding neuropil, will take up the dye and turn red when they are excited at the correct wavelength (820 nm for two-photon imaging) (Figure 7C).
    iii. Healthy cells will not absorb the dye and will, therefore, appear black (Figure 7C).
  d. Approach the black cells that are between 2–5 cell layers (20–100 μm) into the organoid and attempt to create a gigaseal between the pipette and the cell membrane (Figure 7D).

***Note:*** Select a smooth section of the cortical organoid from which to record. We did not preferentially target one specific region; instead, we selected undisturbed regions that did not require the recording pipette to be dragged across large portions of the organoid. We typically targeted the top of the organoid or the edges closest to the recording electrode. It is also helpful to identify patches of hSyn-GFP^+^ cells, if available, and target those regions. Note that assembloids at this stage typically have a necrotic core; it is important not to patch within this region (Figure 7D). However, even two-photon imaging is unlikely to visualize cells at that depth. The necrotic core is surrounded by several layers of healthy cells, followed by a one- or two-cell layer thick outer edge that is not suitable for long-term recordings (Figure 7D). Therefore, aim to patch a cell between the second and fifth cell layers (counting from the surface layer and progressing further into the organoid).

***Note:*** At this stage, use standard whole-cell patch-clamp recording techniques, including proper cell targeting, quick removal of positive pressure to transition to gentle negative pressure, and the application of a hyperpolarized holding potential (−60 mV) to obtain a cell-attached configuration. Gentle pulses of negative pressure should be used to enter the whole-cell configuration. A smooth decay in capacitive current should be observed in response to square current injection, indicating low access resistance.

**CRITICAL:** It should be emphasized that experienced patch-clamp electrophysiologists will probably have the most success with this protocol. It is the view of the authors that an individual attempting to learn patch-clamp electrophysiology should begin with a different, less-challenging system such as acute brain slices from rodents.

31. Once a healthy cell has been patched and a stable recording has been obtained, begin the electrical stimulation protocol to determine the synaptic transmission within the assembloid.
  a. First stimulate with a low level of current delivery (0–5 μA) with one or two pulses.
  b. If there is no response, gradually increase the stimulation intensity until the maximum current delivery of the stimulus isolation unit (SIU) is reached.
  c. Use the lowest amount of current needed to reliably evoke a response.
  d. If no response is obtained when using the maximum amount of current, increase the number of stimulus pulses delivered and increase the stimulus pulse duration from 2 ms to 4–6 ms.

**CRITICAL:** If no synaptic response is obtained when using the maximum current delivery from a commercially available concentric bipolar stimulating electrode across multiple cells from multiple assembloids, using a homemade parallel bipolar stimulating electrode may increase success (Figure 7F).

32. If a homemade bipolar stimulating electrode is required, one can be fabricated as follows (Figure 7F):
  a. Wind two pieces of wire (stainless steel or tungsten) together, leaving the two ends separated by 0.5 mm.
  b. Solder electrical pins to the opposite free ends and secure them with heat-shrink tubing.
  c. Mount the homemade electrode on the electrophysiology rig and connect the pins to the leads attached to the SIU.
  d. Gently insert both prongs of the homemade stimulating electrode into the tissue, taking care to ensure that both prongs are embedded.
  e. Repeat step 31.

***Note:*** The distance between the free prongs can be increased or decreased based on the size of the assembloid; however, it is critical that they are not touching at the ends. Using a wider distance between the prongs can increase the number of cells that are excited within the thalamic organoid, thereby increasing the chances of an electrical signal being transmitted to the cortical organoid.

33. To perform corticothalamic recordings, perform steps 29 through 31 but position the bipolar stimulating electrode in the center of the cortical side of the assembloid and carry out whole-cell patch-clamp recording on the thalamic side.
34. After obtaining a synaptic response (Figure 7E), proceed to deliver a high- or low-frequency stimulation to induce long-term plasticity.

***Note:*** When delivering a high current amplitude, bubbles may form in the organoid after a high-frequency stimulation. If this happens, we recommend that the assembloid not be used for additional electrophysiology experiments.

**CRITICAL:** Aside from spike timing–dependent plasticity protocols, it is imperative that only one cell is collected per assembloid for long-term plasticity experiments.

## Expected outcomes

The protocol enables the generation of thalamocortical assembloids from hiPSC lines. It also describes patch-clamp recording of these assembloids to show that they exhibit synaptic plasticity in response to electric stimulation. In addition to the iPSC lines described here, we have successfully applied this protocol to at least two additional iPSC lines (2242, 8858) that were obtained from the Pasca lab.^54^ In addition to Figure S2 of our published article,^1^ we have unpublished data to support this claim.

When differentiating a thalamic lineage–specific reporter line, such as the TCF7L2-tdT^+^ line, and a cortical lineage–specific reporter line, such as the VGLUT1-tdT^+^ line, by using the protocols described here, high-quality thalamic and cortical organoids can be obtained, albeit with varying efficiency (Figure S1). The thalamic organoids generated here have smooth, translucent edges (Figure 2C). At younger stages, they exhibit low levels of TCF7L2-tdT fluorescence. However, this expression starts to increase at 7 weeks and remains strong for up to 5 months or longer (Figures 2D and 2F). It can even be seen in dying organoids.

Young cortical organoids have multiple neuroepithelial structures that can be seen clearly in bright field, as well as through the expression of tdTomato starting at D40 (Figures 4E and 4F). No tdTomato fluorescence can be detected before D40. As the progenitors of the neuroepithelial structures continue to differentiate into neurons, these structures start to disappear so that by 10 weeks, they can no longer be seen clearly (Figure 4G). At this stage, the organoids are spherical and have smooth and translucent edges (Figures 4C and 4D).

Starting at around D35, both cortical and thalamic organoids tend to expand in size, and they continue to do so until around D90. During this time, routine cutting of organoids is necessary to maintain their size between 500 μm and 1200 μm in diameter (Figure 2E and 4D). This mitigates the level of necrosis in the center of the organoids, alleviates cell death and disintegration of the organoids, and reduces the extent of hypoxia that they experience.^5^^,^^17^ The results of extensive analysis of the cellular composition of these organoids have been reported in our published article.^1^

Depending on the specific hiPSC line used for these experiments, we expect efficiency of up to 90% in generating both thalamic and cortical organoids. However, this efficiency can vary between experiments (Figure 2F and 4E). Because of this experimental variability, the same hiPSC line must be differentiated and assayed multiple times before any conclusions can be drawn. As our organoids are routinely cut to maintain their size, we often obtain a yield that is higher than expected from a single 96V plate.

The thalamocortical assembloids are formed in various sizes and shapes, with varying levels of fusion between the cortical and thalamic halves (Figure 6A). The size and shape of an assembloid do not appear to reflect its maturation level or functionality in electrophysiological assays when the two halves are well fused (Figures 6B and 6C). Poor-quality assembloids may be incompletely fused, show clear signs of cell death and disintegration, have a fluffy appearance, have poor integrity, or lack smooth edges (Figure 6D). Good quality assembloids with smooth edges and a dense morphology are ideal for electrophysiological recordings (Figures 6B and 6C). As early as 7 days post fusion, we have observed axons extending from cortical neurons innervating the thalamic side of the assembloids (Figure 6E). Although the density of this innervation varies between assembloids, we have observed no clear correlation between the density of these axons, as seen on a fluorescence microscope, and the extent of neuronal maturation and synaptic plasticity, as seen during patch-clamp recordings.

## Limitations

Our protocol for generating thalamic organoids is not 100% efficient. Therefore, to ensure consistency and reproducibility of the data, it is imperative that a thalamic lineage–specific reporter line is used for generating thalamic organoids with this protocol. There can be considerable experimental variability in the efficiency of thalamic induction and in the percentage of TCF7L2-tdT^+^ thalamic organoids in the population. In addition, although this protocol can be used to generate thalamic organoids from multiple hiPSC lines, consistent with previous reports in the field, not all hiPSC lines respond to this protocol with equal efficiency.

Our protocol for generating cortical organoids is also less than 100% efficient. Therefore, we strongly recommend using a cortical lineage–specific reporter hiPSC line for generating cortical organoids with this protocol.

When making assembloids, not all plated organoids pairs fuse with each other. The efficiency of this step can range from 50% to 90%. As these fused organoids continue to grow, the efficiency with which healthy assembloids with mature and physiologically functional neurons and synapses are obtained with this protocol is low and can vary between experiments. Assembloids should be screened for healthy appearance and for smooth and dense morphology before being selected for electrophysiological examination (Figures 6B and 6C). However, some assembloids that appear to be healthy (i.e., that are well fused with smooth edges) and comparable in appearance to the “good” assembloids might lack mature neurons firing action potentials or might not respond with synaptic transmission to electrical stimulation.

Future studies using this protocol will be accompanied by efforts to improve the efficiency of this model system.

## Troubleshooting

### Problem 1

Organoids fuse to form large clumps while growing in bioreactors (sometimes seen in steps 5 and 11).

### Potential solution

• Reduce the number of organoids in the bioreactor by splitting the population among multiple bioreactors. We recommend having no more than 50 organoids in one 30-mL bioreactor.
• Screen the population for reporter-expressing organoids and continue to maintain only those in culture; discard the rest. This will reduce the size of the population in the bioreactor.
• Cut the fused organoids in half with fine scissors approximately once a week between D40 and D90 to maintain the size of individual organoids below 1200 μm in diameter.

### Problem 2

Organoids undergo cell death and disintegrate while growing in the bioreactor (sometimes seen in steps 6 and 12).

### Potential solution

• Maintain the size of the individual organoids between 500 μm and 1200 μm in diameter.
• For cortical cultures, supplementing the medium with 0.1% GFR-Matrigel might help mitigate the disintegration of organoids.

### Problem 3

Organoids fail to fuse to form an assembloid in a low-attachment 24-well plate (as seen in step 16).

### Potential solution

• Make sure to select only healthy assembloids for fusion. Both cortical and thalamic organoids must have smooth, translucent edges, and they must have strong integrity. If organoids appear soft and fluffy, they should not be used for assembloid formation.
• Leave plates containing organoid pairs tilted inside the incubator for an additional day or two (5 or 6 days in total).
• Increase the percentage of GFR-Matrigel in the D0 organoid fusion medium (OFM) from 0.5% v/v to 1% v/v.

### Problem 4

iPSCs fail to form EBs or generate cystic-looking EBs in the 96V plate (sometimes seen in steps 3 and 7).

### Potential solution

This could be because of the poor quality of the starting hiPSC line.

• Make sure the starting population of hiPSCs exhibits no spontaneous differentiation and that the colonies are tightly packed.
• Make sure the starting culture is not more than 80% confluent.
• If an hiPSC line repeatedly fails to generate good EBs, it might be best to proceed with a different clone from the same donor.
• If an hiPSC line repeatedly fails to generate good EBs and no alternate clones are available, re-derive new clones from the same hiPSC line. To do this, use FACS to sort the line into single cells in an hES-Matrigel–coated 96-well plate and expand the clones that are tightly packed. Validate the clones (in accordance with our recommendation in the ‘before you begin’ section) before differentiating any of them.
• It is not always possible to explain why EB formation fails. In such cases, repeating the experiment from the start will most likely resolve the issue. If no EB formation is seen by D6, the experiment should be repeated.

### Problem 5

Assembloids start to die 2–3 weeks post fusion (related to step 17).

### Potential solution

This is most likely due to the poor quality of the organoids that were selected for making the assembloid.

• Select smaller organoids (500 μm–900 μm in diameter) for fusion. Larger organoids have a greater tendency to undergo cell death post fusion.
• Make sure the organoids are dense and have smooth and translucent edges. Organoids that appear soft or fragile should not be used for making assembloids.

### Problem 6

The efficiency of thalamic induction is low, leading to a low percentage of TCF7L2-tdT^+^ organoids in the population.

### Potential solution

This could be because the hiPSC line is of poor quality or has low neurogenic potential.

• Make sure the starting population of hiPSCs exhibits no spontaneous differentiation and that the colonies are tightly packed.
• Make sure the starting culture is not more than 80% confluent.
• If an hiPSC line repeatedly generates thalamic organoids at low efficiency, it might be best to proceed with a different clone from the same donor.
• If an hiPSC line repeatedly generates thalamic organoids at low efficiency and no alternate clones are available, re-derive new clones from the same hiPSC line. To do this, use FACS to sort the line into single cells in an hES-Matrigel–coated 96-well plate and expand the clones that are tightly packed. Validate the clones (in accordance with our recommendation in the ‘before you begin’ section) before differentiating any of them.

### Problem 7

The efficiency of cortical induction is low, leading to a low percentage of VGLUT1-tdT^+^ organoids in the population.

### Potential solution

This could be because the hiPSC line is of poor quality or has low neurogenic potential.

• Make sure the starting population of hiPSCs has no spontaneous differentiation, and that the colonies are tightly packed.
• Make sure the starting culture is not more than 80% confluent.
• If an hiPSC line repeatedly generates cortical organoids at low efficiency, it might be best to proceed with a different clone from the same donor.
• If an hiPSC line repeatedly generates cortical organoids at low efficiency and no alternate clones are available, re-derive new clones from the same hiPSC line. To do this, use FACS to sort the line into single cells in a hES-Matrigel coated 96-well plate and expand clones that are tightly packed. Validate the clones (in accordance with our recommendation in the ‘before you begin’ section) before differentiating any of them.

## Resource availability

### Lead contact

Further information and requests for resources and reagents should be directed to, and will be fulfilled by the lead contact, Stanislav S. Zakharenko (stanislav.zakharenko@stjude.org).

### Technical contact

Anjana Nityanandam (anjana.nityanandam@stjude.org).

### Materials availability

This study did not generate any new, unique reagents.

### Data and code availability

The published article includes all datasets generated or analyzed during this study.

## Acknowledgments

We thank Lawrence Reiter for providing the TP-190a dental pulp stem cells, the Center for Advanced Genome Engineering (CAGE) at St. Jude for generating reporter hiPSC lines, the Cytogenetics Core at St. Jude for karyotyping, the Hartwell Center at St. Jude for DNA methylation assay, Keith Laycock for manuscript editing, and Zakharenko lab members for constructive comments. This work was funded, in part, by National Institutes of Health grants R01MH097742 and R01DC012833 (to S.S.Z.) and K99MH129617 (to M.H.P.), Stanford Maternal and Child Health Research Institute Uytengsu-Hamilton 22q11 Neuropsychiatry Research Program grants UH22QEXTFY21 and UH22QEXTFY23 (to S.S.Z.), National Cancer Institute grant P30CA021765, and the American Lebanese Syrian Associated Charities (ALSAC). The content is solely the responsibility of the authors and does not necessarily represent the official views of the National Institutes of Health or other granting agencies. All figures were created in BioRender.

## Author contributions

Conceptualization, A.N., M.H.P., K.T.T., and S.S.Z.; hiPSC line maintenance, organoid differentiation, and assembloid preparation, A.N. and K.D.N.; electrophysiology, M.H.P. and I.T.B.; validation, K.T.T.; visualization, A.N.; funding acquisition, S.S.Z.; supervision, S.S.Z.; writing – original draft, A.N., M.H.P., and K.D.N.; writing – review and editing, K.T.T. and S.S.Z.

## Declaration of interests

The authors declare no competing interests.
