# Supplementary material for: Protocol for generating human assembloids to investigate thalamocortical and corticothalamic synaptic transmission and plasticity
Source: STAR Protoc. 2025 Feb 7;6(1):103630. doi: 10.1016/j.xpro.2025.103630 (PMC11850219; doi:10.1016/j.xpro.2025.103630)
Supplement: Document S1. Figure S1 [file mmc1.pdf]

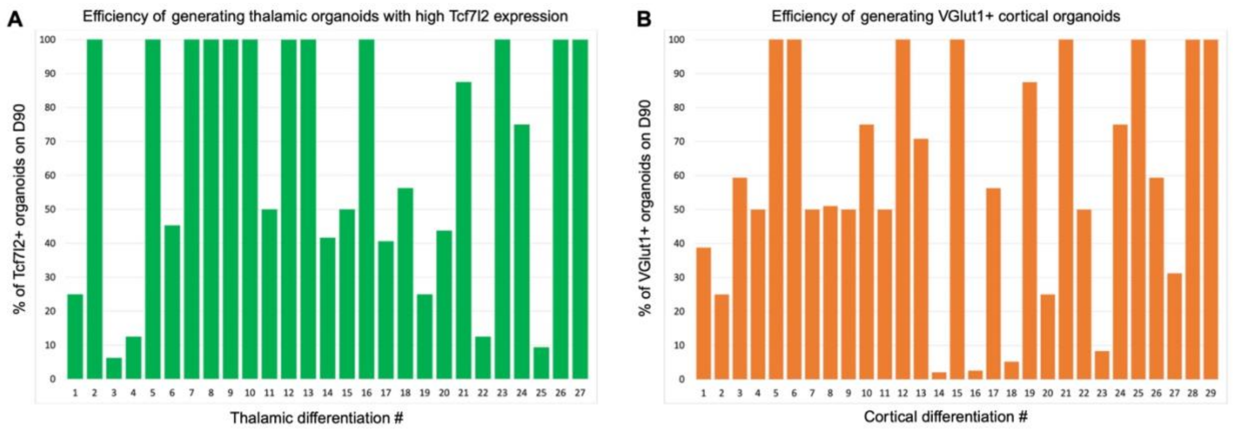

Figure S1. Quantification of experimental variability in organoid generation.

(A) Efficiency of generating thalamic organoids with high Tcf7l2 expression in 27 independent differentiations using the Tcf7l2-tdT⁺ iPSC line.
(B) Efficiency of generating cortical organoids with high VGlut1 expression in 29 independent differentiations using the VGlut1-tdT⁺ iPSC line.
